# Against the grain: International migrants, the children of migrants and national life expectancy in Sweden, 1990–2019

**DOI:** 10.1016/j.ssmph.2024.101726

**Published:** 2024-11-08

**Authors:** Matthew Wallace, Sven Drefahl

**Affiliations:** a Centre for Research on Inclusive Society, School of Health and Society, University of Salford, Frederick Road Campus, Broad Street, Salford, M6 6PU, United Kingdom; b Stockholm University Demography Unit, Stockholm University, Universitetsvägen 10B, Stockholm, S106-91, Sweden

**Keywords:** Migrants, Children of migrants, Mortality, National life expectancy, Population health, Demography

## Abstract

International migrants and their children represent increasing shares of the populations of major host countries and have growing potential to affect estimates of national mortality. Yet, while many studies have observed mortality differences between migrants, their children, and the majority population, few have progressed beyond this point to quantify the actual impact of these differences upon national life expectancy levels. Studies that have, reveal that migrants increasingly enhance national life expectancy, but do not progress beyond a single average generational effect. Here, using established demographic methods, we aim to quantify and unpack the impact of migrants and the children of migrants on national life expectancy in Sweden, with emphasis on potential differences by age, generations, and migration background. Going “against the grain” relative to other countries, we reveal an initial negative effect of first-generation migrants on national life expectancy levels in Sweden, followed by a gradual waning and disappearance of this effect over time. This change is attributable to the transformation in origin composition of Sweden's migrant population from migrants born in Nordic countries (that have higher mortality than the majority population) to migrants born in non-Western countries (that have lower mortality than the majority population), particularly at working ages. For children of migrants, nearly all ages and migrant backgrounds contribute to an increasingly negative effect on national life expectancy over time. The unique and disparate mortality risks of migrants, the children of migrants, and the majority population suggest a need to monitor their mortality separately so as to maximise potential future gains in national life expectancy in Sweden.

## Highlights

• The effect of migrants on life expectancy shifted from negative to positive over time.
• This turnaround was driven primarily by young adult non-Western migrants.
• The effect of the children of migrants on life expectancy is negative.
• This was driven by mortality among children of Nordic and non-Western migrants.
• Negative second-generation effects were concentrated in infancy and working ages.

## 1 Introduction

International migrants (the *first-generation*) and their children, who are born in the host country that migrants move to (the *second-generation*), comprise substantial and growing shares of the resident populations of major host countries. In 2014,^1^ one in five residents of countries of the European Union (EU) were first or second-generation (Agafiţei & Ivan, 2016). This proportion is even higher in major immigrant-receiving countries like Belgium, France, Sweden, and the United Kingdom, where the proportions are closer to one in three (Agafiţei & Ivan, 2016). In the United States (U.S.), the equivalent value is closer to one in four (Batalova et al., 2022). Today, the potential for migrants and their children to exert lasting demographic change in the host country in terms of its population size, rate of change, composition, age structure, and its fertility and mortality levels is greater and more dynamic than ever before (Coleman, 2008).

In recent decades, an impressive number of articles have studied mortality differences between migrants and non-migrants (*see recent reviews by* Aldridge et al., 2018; Shor & Roelfs, 2021) and the children of migrants and the children of non-migrants (*see the recent review by* Wallace et al., 2023). Their meta-analyses tell us that the global risk of mortality is *lower* among migrants compared to non-migrants born in the host country (the so called ‘migrant mortality advantage’) (Aldridge et al., 2018; Shor & Roelfs, 2021), but *higher* among children of migrants compared to children of non-migrants in Europe (Wallace et al., 2023), especially in young to middle adulthood (Aldridge et al., 2018; Shor & Roelfs, 2021; Wallace et al., 2023). Of course, there is heterogeneity in both the size and direction (i.e., higher or lower risks than non-migrants) of mortality risk according to factors such as origins, age, and—for migrants—age at arrival and duration of residence (Aldridge et al., 2018; Shor & Roelfs, 2021; Wallace et al., 2023).”

Unlike other demographic consequences of migration, the mortality of migrants and their children is considered a niche topic that is disconnected from the wider mortality situation of the host country they live in, rather than something that might prove central to understanding national patterns and trends in mortality (Acevedo-Garcia et al., 2012). The combination of **(a)** sizeable and increasing numbers of migrants and their children living in major host countries (United Nations Department of Economic and Social Affairs, 2019), **(b)** the ageing-in-place of migrant populations towards older ages of increased mortality risks (Wallace, 2023), and **(c)** the unique mortality risks of migrants and their children at different stages of the lifecourse (Aldridge et al., 2018; Shor & Roelfs, 2021; Wallace et al., 2023) suggest the growing potential of these groups to affect national mortality estimates. It is necessary to ‘re-contextualise’ the mortality of migrants and their children within the wider mortality of national populations. Not least to understand how international migration has affected mortality measures used to monitor national progress in health attainment that underlie myriad public health policies (Hiam et al., 2022).

Our **aim** is to quantify and to decompose the impact of migrants and the children of migrants on national mortality levels in Sweden over a long period of time (1990–2019), with a dedicated emphasis on how this impact varies by (parental) country of birth, sex, age, and all combinations thereof. In line with a small number of existing studies to have done this—which we summarize and evaluate in the background section (Hendi & Ho, 2021; Mehta et al., 2016; Page et al., 2007; S. H. Preston & Elo, 2014; Wallace et al., 2022)—we will use period life expectancy (PLE) as our mortality measure. PLE is defined as the average number of additional years a person of a given age would live if age-specific mortality rates were to remain the same for the remainder of their life. PLE is one of the world's most widely used population health metrics to summarize, compare and rank the current mortality situation of countries, forming the basis for many public health, life insurance and retirement policies (Luy et al., 2020). This is because a country's life expectancy accurately reflects, among other things, its socioeconomic conditions and the quality of its social, welfare, and public healthcare infrastructure (Ho & Hendi, 2018). PLE is conventionally calculated by gender. However, it can also be disaggregated for specific population subgroups. This kind of disaggregation allows for nuanced analyses of PLE, for example among groups with distinct migration backgrounds. We will answer three research questions.

RQ1. What is the average effect of the first-generation and second-generation upon national PLE in Sweden in 1990 and how does this effect change over time?
RQ2. At what ages do the first-generation and second-generation have the biggest effect upon national PLE and how does this age effect change over time?
RQ3. How do different (parental) origins of the first-generation and second-generation affect national PLE in Sweden and does the effect of specific origins change over time?

Sweden represents a fascinating context within which to conduct this piece of research. Together, the first-generation (19.6%) and second-generation (11.2%) accounted for around 31%^2^ of the total population of Sweden in 2014 (Agafiţei & Ivan, 2016). This is one of the highest proportions in all of Europe and these proportions have grown substantially over time. Moreover, Sweden is one of the most diverse societies in the European Union (EU) (Schierup & Ålund, 2011). Three in four first-generation migrants were born outside of the EU (Agafiţei & Ivan, 2016), owing to Sweden's liberal refugee policy (Karlsdottir et al., 2018). Sweden's long migration history means that a substantial share of migrants have reached older ages and that a second-generation has been well-established (Pedersen et al., 2008). In all, Sweden offers large and diverse first and second-generations that have the potential to affect national mortality estimates.

## 2 Background

### 2.1 Previous empirical findings

Few studies have estimated the effect of the unique mortality patterns of migrants and their children on national population health metrics like PLE. We summarize those that have below.

Perhaps the first study to do this originated from Australia, where migrants were shown to increasingly enhance national life expectancy at birth over time. Specifically, the contribution of migrant men increased from 0.3 years to 0.6 years between 1983 and 2001; the contribution of migrant women remained constant at around 0.4 years (Page et al., 2007). The author concluded that an increase in the number of migrants since the 1950s as a proportion of the total Australian resident population, had resulted in an even greater increase in national life expectancy than would otherwise have been anticipated. The effect, they reasoned, was not trivial and had been obscured, as only the total resident population life expectancy is routinely reported (Page et al., 2007).

In the U.S., Preston and Elo (2014) investigated the potential causes of rapid gains in life expectancy at birth in New York between 1990 and 2010. In 1990, life expectancy at birth in New York was 4.27-years lower among men and 1.80-years lower among women compared to the national average. Over the next two decades, however, life expectancy at birth in New York surged and, by 2010, was 1.73 years *higher* among men and 2.06-years *higher* among women compared to the U.S. national average. The authors examined migrant status as one potential important explanation. When they compared the life expectancy at birth of non-migrants and migrants living in New York to their respective U.S. averages in 2010, the authors found the longevity of non-migrants living in New York versus all the U.S. (78-years) and migrants living in New York versus all the U.S. (83-years) to be identical. The key difference that caused such a surge in life expectancy in New York was the considerably higher proportion of international migrants residing in New York (37.8%) compared to the U.S. average (13.6%) ( Preston & Elo, 2014).

Mehta et al. (2016) focused upon life expectancy at age 65+ in the U.S. Alongside seeing 2.38-year and 2.37-year advantages in life expectancy at age 65 among first-generation men and women compared to U.S.-born men and women in 2000-9, the paper showed a contribution of 0.22 years among migrant men and 0.23 years among migrant women to national U.S. life expectancy at age 65. The authors reasoned that this contribution was not trivial given the years of life left (Mehta et al., 2016). Although the individual impact of specific migrant origins was not estimated, life expectancy at age 65 was higher among migrants from all different origins compared to the U.S-born. This ranged from 4.5 years higher among male migrants from South Central Asia to 1-year higher among male migrants from Canada and 3.5 years higher among female migrants from South America to 1-year higher among Oceanian migrants (Mehta et al., 2016).

The most recent U.S. study from Hendi and Ho (2021) found that immigrants increasingly enhanced life expectancy at age 1 between 1990 and 2017, with their contribution rising from 0.32 years to 0.94-years for men and from 0.26-years to 0.83-years for women (Hendi & Ho, 2021). While the relative proportion of migrants has increased in this time, so did their longevity advantage. Specifically, the life expectancy at age 1 of migrant men was 4-years higher in 1990 but 6.5-years higher in 2017. Meanwhile, the life expectancy at age 1 of migrant women was 3-years higher in 1990 but 5.7-years higher in 2017 (Hendi & Ho, 2021). When incorporating the second-generation (i.e., children of migrants), the contributions became even larger. This finding represents a dissonance between the mortality situation of the children of migrants in the United States (where their mortality risks, much like the first-generation, tend to remain somewhat lower than those of non-migrants) compared to Europe (where the mortality risks of the children of migrants are higher compared to both migrants *and* non-migrants (Wallace et al., 2023). A combined first-generation and second-generation men contributed 0.65–1.20-years to life expectancy at age 1 in 2000 to 1.22–1.45-years in 2017. A combined first-generation and second-generation women contributed 0.67–1.20 years to national life expectancy at age 1 in 2000 to 1.23–1.25-years in 2017 (Hendi & Ho, 2021). When decomposing the contributions of a combined first-generation and second-generation by age, the authors found that the bolstering effect of migration on life expectancy at age 1 was concentrated at ages 25–64 (Hendi & Ho, 2021).

Finally, a comparative study from the Nordic region documented an enhanced effect of migrants on life expectancy at age 1-year in Denmark, Finland, and Norway, but not in Sweden between 1990 and 2019. For Sweden, the authors found an initial *negative* impact of migrants upon national life expectancy of −0.19 years in the early 1990s. The size of this negative impact in Sweden reduced over time and disappeared by 2019. This transformation reflected a faster pace of increase in life expectancy at age 1-year among migrants over time. For example, life expectancy at age 1-year among male migrants was 1.6-years lower than native-born in 1990 (72.7-years among migrants versus 74.3 among native-born) but virtually identical to native-born by 2019 (80.5 years among both migrants and native-born) (Wallace et al., 2022). For men in Finland and Denmark, and men and women in Norway, migrants were shown to increasingly enhancing national life expectancy over time. The size of the effect was largest among migrants in Norway in recent years, with peak impacts of +0.19 years among men and +0.18 years among women, followed by men in Finland (+0.16 years). For men in Denmark, the size of effect was +0.09 years. Such contributions reflected higher, but not necessarily increasing, life expectancy at age 1-year among migrants compared to native-born. The authors conclude that, although the size of the effects were modest, the gradual growing impact of migrants upon life expectancy over time was clear (Wallace et al., 2022). Moreover, they found that these effects were having an impact upon rankings of national life expectancy within the Nordic region. Specifically, by accelerating gains in national life expectancy among men in Norway relative to men in Sweden and among women in Norway and Finland as compared to women in Sweden. (Wallace et al., 2022).

In summary, these findings reflect the positive effect of a pervasively observed “migrant mortality advantage” (i.e., a lower mortality risk among immigrants when compared to non-migrants) and are important in showcasing the tangible impact of immigrants on national population mortality. Nevertheless, nearly all the studies produce average generational effects that combine a highly disparate range of migrant origins (Hendi & Ho, 2021; Page et al., 2007; Preston & Elo, 2014; Wallace et al., 2022). We know that mortality risks (relative to non-migrants) vary considerably by country of birth. Lower mortality risks have only been consistently reported among migrants born in non-Western countries living in Western countries. Migrants born and living in Western countries have weaker to non-existent, mortality advantages, or even mortality disadvantages, compared to non-migrants (Shor & Roelfs, 2021). The same average generational effect also applies to age (Page et al., 2007; Preston & Elo, 2014; Wallace et al., 2022). Mortality among migrants compared to non-migrants does not remain proportionate across the lifecourse. It is higher in infancy, childhood and adolescence, lower in young to mid-adulthood, and increasingly similar to, or higher, than non-migrants at older adult ages (Guillot et al., 2018; Kobori et al., 2017; Shor & Roelfs, 2021; Trovato & Odynak, 2011; Wallace & Wilson, 2022). These average generational effects *must* mask substantial heterogeneity in the impact of migrants on national mortality according to age and country of birth. Only one study examines the effect of the second-generation. Yet, it does not offer an explicit estimate, instead producing a single value for a combined first and second-generation population (Hendi & Ho, 2021). However, a recent review suggests that mortality risks of the first-generation and second-generation in Europe diverge, with much lower mortality risks in the first-generation and higher mortality risks in the second-generation compared to non-migrants (Wallace et al., 2023). Last, the clear exception from the studies is Sweden, where migrants depress national life expectancy up until recently. However, beyond a single generation-level estimate, we know little else about this case.

### 2.2 The Swedish case

Having previously been a nation of *emigration*, Sweden was transformed into a nation of *immigration* in the 1940s by the arrival of European refugees during the Second World War (Migrationsverket, 2020). After the end of the war, Sweden began to receive increasingly large numbers of labour migrants—predominantly from Finland (Migrationsverket, 2020). This was driven by agricultural decline and rising unemployment in Finland, combined with national economic growth and strong demand for unskilled labour in Sweden (Korkiasaari & Söderling, 2003). This large-scale in-flow was facilitated by the 1954 *Nordic Common Labour Market* agreement that permitted free movement within the Nordic region (Hedberg & Kepsu, 2003). Simultaneously, there was *some* migration from outside of the Nordic region at this time too, with smaller inflows of labour migrants from countries like Greece, Turkey, and Yugoslavia (Bevelander et al., 2013). Unlike other recruiting countries at that time, which pursed a “guest worker” policy, Sweden's government assumed migrants would stay, integrate and become full citizens. Following the implementation of an official “immigration stop” in 1972 as the national economy slowed, these inflows were replaced by different forms of migration. This included the arrival of many family members of labour migrants already living in Sweden (Borevi, 2014). It also included sizeable humanitarian migration flows and the beginning of a regular stream of refugees from low and middle-income countries. Notably, the arrival of refugees from Chile and Lebanon (1970s), Iran, Iraq, Lebanon, and Eritrea (1980s) and former Yugoslavia, Somalia and Ethiopia (1990s) (Migrationsverket, 2020). In 2001, Sweden's Schengen membership led to increasing numbers of European Union (EU) migrants moving to Sweden (particularly from those countries that were part of the 2004 and 2008 EU accession agreements) in order to purse higher education and/or labour opportunities (Migrationsverket, 2020). The 2010s ‘European migrant crisis’ led to the arrival of a large number of refugees from Syria (Migrationsverket, 2020).

## 3 Material & methods

### 3.1 Data

We use the collections of administrative register data “*Ageing Well*” held at Stockholm University. This data is accessible for research under ethical approval from the Swedish Ethical Review Authority. "*Ageing Well*” comprises pseudonymised linked, longitudinal micro-level data from several administrative registers that covers the total population of Sweden from 1968 to 2019. To compare with Wallace et al. (2022), we focus on years 1990–2019 and use data from the total population register, migration register, death register, and the multigenerational (parent-child) register. We use the following variables: sex, year of birth, individual country of birth, parental country of birth, year of death, and being registered as resident in a given year. From these variables we further derive exact age, the exact age-at-death, and broad generational status.

The first-generation (G1) are defined as people born in any country other than Sweden, the second-generation (G2) as people born in Sweden to at least one parent born abroad and the majority population as people born in Sweden to two parents born in Sweden. We further define first and second-generation with Nordic, Western (and non-Nordic background), and non-Western origins. Supplementary Box S1 details how the most detailed level countries/regions of birth available to us in our data are organised into these three categories. Supplementary Table S2 and Supplementary Table S3 showcase how the composition of these three categories has transformed over time according to more detailed (parental) origins and age. Supplementary figures S6, S7 and S8 explicitly examine the role of having one versus two migrant parents on our results. These supplements are discussed in the section ‘Supplementary analyses’ at the end of the results section. For a small number of cases where a second-generation person has two migrant parents with different origin country backgrounds (e.g., a parent with a Western background and a parent with a non-Western background) we assign those cases to the birth region of the mother.

### 3.2 Methods

We first collapse the register data into an aggregated format for deaths and population sizes by year (1990–2019), age (in single years from 0-1-years old to open-ended interval 95-years+), sex (male and female), generation (total population, majority population, first-generation, and second-generation), and generation by origins (majority population, G1 Nordic, G1 other Western, G1 non-Western, G2 Nordic, G2 other Western, G2 non-Western). For deaths, in a calendar year we calculate exact age-at-death (i.e., $\frac{date\;of\;death-date\;of\;birth}{365.25}$), create a death indicator (i.e., 1 = died, 0 = alive), and aggregate the deaths by age, sex, and migrant background.

For population sizes, we create a dichotomous variable that indicates residence or not in each country at the end of a calendar year (i.e., 1 = resident, 0 = not resident) according to age, sex, and generational status. Whether or not someone is resident is determined using trace evidence from multiple registers (Maret-Ouda et al., 2017). From the population counts, we then calculate midyear population estimates ($\frac{\sum people\;aged\;x\;in\;year\;t+\sum people\;aged\;x\;in\;year\;t+1}{2}$), which provide an indication as to how many people are living in a country during a calendar year, accounting for births, deaths and migration events. We calculate mid-year estimates, and not person-years, to maximise the comparability of our estimates with those of Wallace et al. (2022).

We calculate age-specific death rates by sex and nativity status by dividing the death counts by the midyear estimates (i.e., $\frac{deaths\;at\;age\;x\;in\;year\;t}{midyear\;population\;at\;age\;x\;in\;year\;t}$). The age-specific death rates and midyear estimates are fed into R package Demography (see (Hyndman et al., 2019)) to generate period lifetables. The calculations forming the basis of the lifetable function in R package Demography can be seen in Chiang, 1984; Keyfitz & Caswell, 2005; Preston et al., 2001.

We generate lifetables and period life expectancy at birth (PLE*0*) for **(a)** the total population, **(b)** the majority population, and **(c)** G1 *and* G2 combined. Lifetables are closed at 95+.

To first quantify the impact of the first-generation and second-generation upon national population mortality we look at the gap in life expectancy between the total population and the majority population. This is consistent with research to have studied the impact of the mortality of migrants on national mortality (Hendi & Ho, 2021; Page et al., 2007; Preston & Elo, 2014; Wallace et al., 2022).^3^ We note, however, that while we use PLE0, two studies use PLE1 (Ho & Hendi, 2018; Wallace et al., 2022). For a detailed discussion and justification as to why we use PLE0 for our particular study, and not PLE1, please refer online to Supplementary Box S2.

Then, we decompose the gap between the life expectancy at birth of the total population and the life expectancy at birth of the majority population by increasingly detailed combinations of generation, age, and origin using the standard *Arriaga* decomposition method (Auger et al., 2014). Supplementary Box S3 provides a description—and the formulas—associated with this method.

From a potential starting sample of 13,862,633 people who were registered as resident at least one year in Sweden between 1989 and 2019, we had to exclude 137,533 people who had a missing country of birth (0.99%) who could not be categorised as majority population, first-generation, or second-generation. This left a final sample of 13,725,100. Although we *suspect* that a substantial share of this 137,533 people is foreign-born (e.g., 101,815 [74%] had at least one registered immigration date and many of them cannot be linked to any parents in the multi-generational register) it is not possible to state definitively if they are first-generation migrants. Yet, even if *all* of them were first-generation, this group would still only constitute 4.7% of all migrants. Sensitivity analyses that (1) model the PLE of this group explicitly, (2) include this group as part of the first-generation, and (3) exclude this group as part of the first-generation demonstrate no tangible effect on the final set of results we present below (that excludes this group).

## 4 Results

### 4.1 Descriptive results

Table 1 displays the population sizes at the generational-level and shows how they have changed over time. Absolute and relative numbers of first and second-generation have increased over time from 802,659 (9.3%) and 622,529 (7.2%) respectively in 1990 to 1,919,083 (18.8%) and 1,373,637 (13.5%) in 2019. Simultaneously, absolute and relative numbers of the majority population have continuously decreased over time, meaning that continued population growth in Sweden is being sustained by migrants and their children. These patterns and trends show that the first-generation and second-generation will exert growing weight in national mortality estimates.

**Table 1S:** Changes in absolute and relative population sizes the majority population, first-generation, and second-generation in Sweden, 1990–2019.

Year	Population size (n)	Proportion of total resident population (%)	% change from the time period before
Majority population			
1990	7,164,663	83.4	–
1995	7,159,490	81.0	−0.1
2000	7,048,347	79.4	−1.6
2005	6,985,464	77.2	−0.9
2010	6,953,998	73.9	−0.5
2015	6,929,992	70.3	−0.3
2019	6,910,697	67.7	−0.3
First-generation			
1990	802,659	9.3	–
1995	948,391	10.7	+18.2
2000	1,016,233	11.4	+7.2
2005	1,137,691	12.6	+12.0
2010	1,398,446	14.9	+22.9
2015	1,689,594	17.2	+20.8
2019	1,919,083	18.8	+13.6
Second-generation			
1990	622,529	7.2	–
1995	728,404	8.2	+17.0
2000	816,836	9.2	+12.1
2005	922,698	10.2	+13.0
2010	1,063,064	11.3	+15.2
2015	1,231,395	12.5	+15.8
2019	1,373,637	13.5	+11.6
Total resident population			
1990	8,589,851	100.0	–
1995	8,836,285	100.0	+2.9
2000	8,881,416	100.0	+0.5
2005	9,045,853	100.0	+1.9
2010	9,415,508	100.0	+4.1
2015	9,850,981	100.0	+4.6
2019	10,203,417	100.0	+3.6

**Fig. 1:**
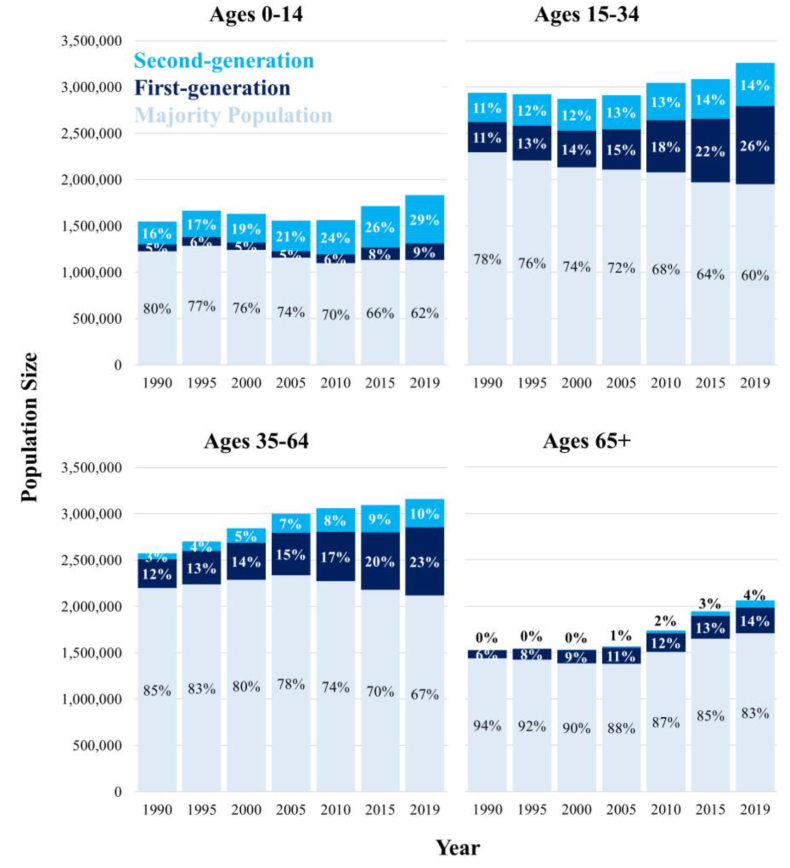
The changing composition of Sweden's population over time by age and generational status, 1990–2019. Source: authors' calculations based upon Swedish register collection “Ageing Well”.

Fig. 1 showcases the increase in both the absolute and relative proportions of the first and second-generation across the age groups 0–14 years, 15–34 years, 35–64 years and 65+ years. The share of first-generation is largest, and has increased the most, in age groups of 15–34 years (from 326,331 [11%] in 1990 to 840,633 [26%] in 2019) and 35–64 years (from 309,414 [12%] in 1990 to 734,711 [23%] in 2019). The share of second-generation is largest and has increased the most in the age group 0–14 years (242,737 [16%] in 1990 up to 524,980 [29%] in 2019). The first-generation will carry the largest weight in national mortality estimates at ages 15–64 years, while the second-generation will carry the greatest weight at ages 0–14 years.

**Fig. 2:**
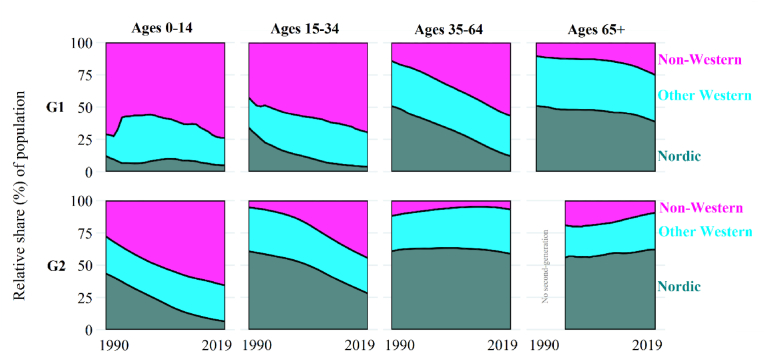
The changing origin composition of Sweden's first-generation and second-generation populations over time and age, 1990–2019. Source: authors' calculations based upon Swedish register collection “Ageing Well”.

Fig. 2 reveals how the country of birth composition of the first-generation and the parental country of birth composition of the second-generation have transformed over time in the same age bands as shown in Fig. 1. For the first-generation, the most significant changes in origin composition are observed in the age groups of 15–34 years and 35–64 years. In these groups, the percentage of migrants born in non-Western countries has risen sharply, from 42% to 70% for the 15–34 age group and from 14% to 57% for the 35–64 age group, from 1990 to 2019. Simultaneously, the percentage of migrants born in other Nordic countries in these groups has declined over time, from 34% to 4% for the 15–34 age group and from 51% to 12% for the 35–64 age group. The youngest age group (0–14 years of age) has remained predominantly non-Western (71% in 1990; 74% in 2019). The oldest age group (65+ years), conversely, has stayed majority Western (90% Nordic and other Western in 1990; 75% Nordic and other Western in 2019).

For the second-generation, the most significant change observed in Fig. 2 occurs within the two youngest age-groups, 0–14 years and 15–34 years. The percentage of second-generation individuals with at least one parent born in a non-Western country has risen sharply over time, from 28% in 1990 to 66% in 2019 for the 0–14 age group and from 5% in 1990 to 45% in 2019 for the 15–34 age group. In the two oldest age groups, 35–64 years and 65+ years, second-generation individuals with at least one parent born in a Nordic country consistently account for just over half of the entire second-generation population at those ages. Consequenrtly, first and second-generation with non-Western origins should carry a large and increasing weight at younger ages over time, while first and second-generations with Nordic origins should carry a large, but decreasing, weight in the mortality of the first and second-generations over time in Sweden.

#### 4.1.1 Main results

**Fig. 3:**
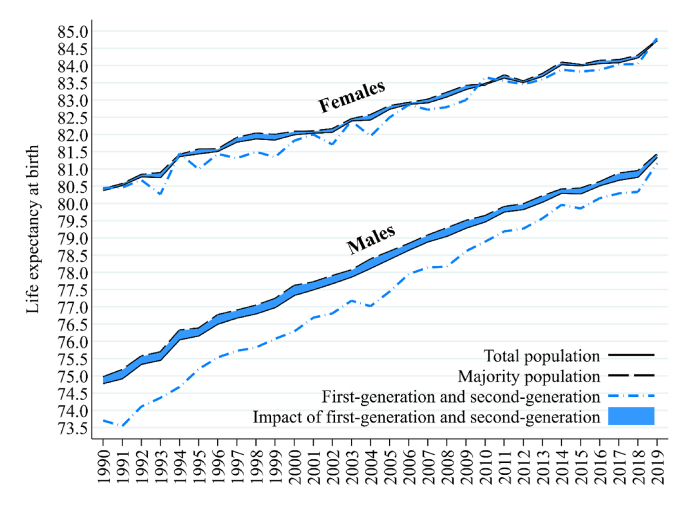
Life expectancy at birth in Sweden with and without the first and second-generation, 1990–2019. Notes: the total population minus first and second-generation is the life expectancy of the majority population (i.e., born in Sweden to two parents born in Sweden). Source: authors' calculations based upon Swedish register collection “Ageing Well”.

Fig. 3 shows long-term trends in life expectancy at birth (PLE*0*) in Sweden from 1990 to 2019. For the total populations of men and women in Sweden, PLE*0* has steadily increased over time, closely in line with official national statistics (Statistiska Centralbyrån, 2021). For men within the majority population (or alternatively, PLE*0* in the absence of the first-generation and the second-generation populations), PLE*0* is higher (74.95 years) than for the total male population in Sweden in 1990 (74.79 years). Over time, the gap between the two measures reduces until they nearly fully converge by 2019, with 81.42 years for majority population men versus 81.35 years for the total male population. The initial gap in 1990 is attributable to the fact that the PLE*0* of the first-generation and the second-generation is over 1.25-years lower, at 73.70 years, than that of majority population men in 1990 (74.96 years). This gap closes at an average pace of 12 days per year, resulting in a smaller gap of 0.26 years by 2019 (81.16 years for first-generation and second-generation men compared to 81.42 years for majority population men).

For women in Fig. 3, the patterns and trends are less defined. It can be observed that the PLE*0* for women within the majority population remains somewhat higher than that of the total female population of Sweden, and the PLE*0* of first-generation and second-generation women is generally lower than that for women of the majority population. Yet, for the most part the three lines track each other closely over the thirty-year period. By 2019, the PLE*0* of all three populations are remarkably similar: 84.74 years for the total population of women, 84.72 years for majority population women, and 84.79 years for first and second-generation women.

**Fig. 4:**
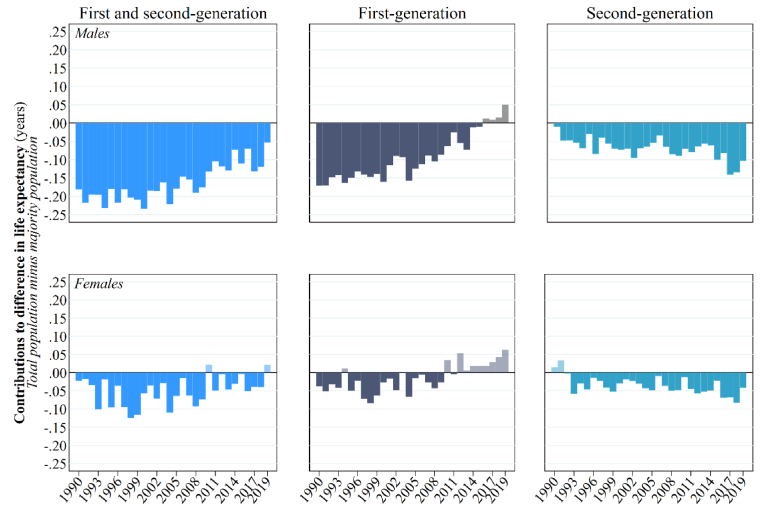
The impact of the first and second-generation upon national life expectancy in Sweden, 1990–2019. Source: authors' calculations based upon Swedish register collection “Ageing Well”.

Fig. 4 displays the impact of the first and second-generation upon national life expectancy in Sweden. It does so by showing the gap between the PLE0 of the total population and total population minus a combined first-generation and second-generation (i.e., the majority population), alongside a decomposition of the gap by generation. In the 1990s, a combined first-generation and second-generation men depress PLE*0* by a fifth to a quarter of year. The effect is largest in 1994, 1996, and 2010 (−0.23 years or 85 days). This negative effect gradually decreases over time to 0.05 years (or 19 days) by 2019. When the difference is decomposed into generations, nearly all the negative impact in the 1990s is attributable to first-generation men (between −0.13 years and −0.17 years through this decade). However, the size and direction of the contribution of first-generation men decreases and reverses over time to the point where they enhance PLE*0* by a negligible amount by 2019 (+0.05 years). The opposite is true for second-generation men, whose small negative effect in 1990 (of around −0.05 years) gradually rises over time to −0.10 to −0.14 years by the late 2010s. The same patterns and trends apply to first-generation and second-generation women, albeit the levels are lower and the trends less defined. At least in the 2010s, first-generation women have a (negligible) positive impact upon PLE*0*.

**Fig. 5:**
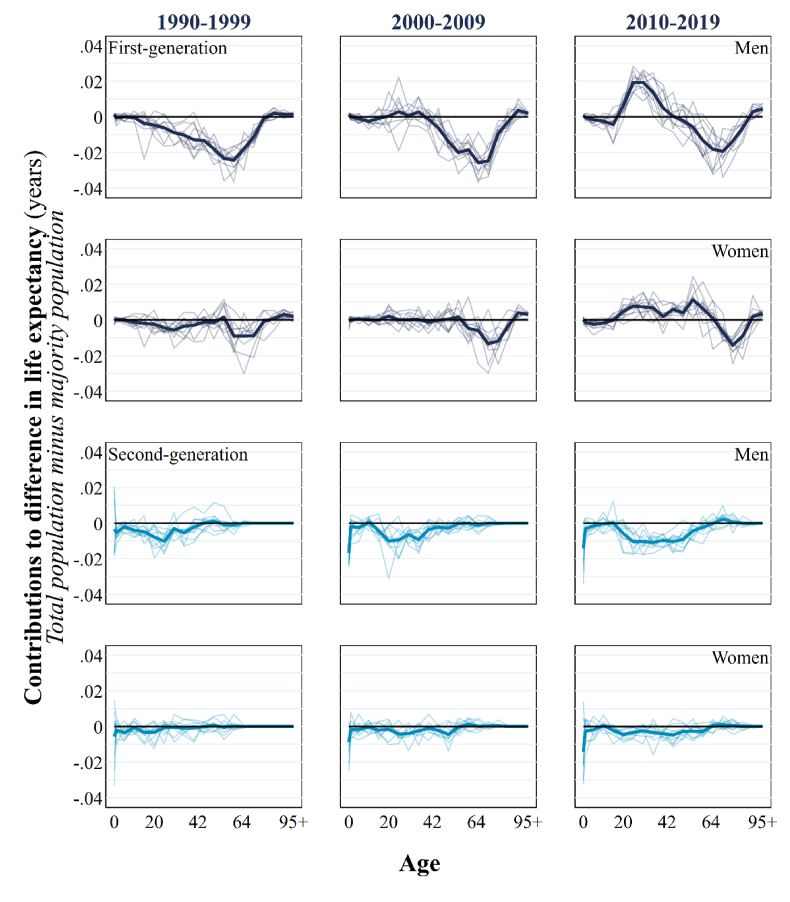
Generation by age decompositions of life expectancy differences between total population and total population minus the first and second-generation, 1990–2019. Source: authors' calculations based upon Swedish register collection “Ageing Well”. Notes: the thick lines in each panel represent an age decomposition for the entire decade; the thin lines represent the annual age decompositions in each decade.

Fig. 5 displays a decomposition in the difference in national PLE*0* and the PLE*0* of the majority population by generation and age. Among first-generation men and women, we see a reversal in the contribution of migrants to PLE*0* from a negative contribution to a positive contribution over time, between the 1990s and 2010s, in young to middle adulthood for ages 20–49 years for men and ages 20–64 years for women. At older adult ages among the first-generation, we see a persistent negative contribution. However, this negative contribution shifts increasingly further up the age range and compresses with each passing decade. These patterns and trends likely reflect a complex combination of factors associated with different time effects. These include age (*senescence*), period (and *emigration policy* in birth countries; *immigration* policy in Sweden), birth cohort (and *variations in exposure to distributions in disease*), arrival cohort (*country of birth* and the extent of epidemiological, political, sociocultural differences to Sweden), age-at-arrival (and the strength of *health in-selection effects* [which are weaker or non-existent in children] and *reason for arrival* [many migrants arriving in Sweden as children are/were refugees), and length of stay (and accelerated ageing among migrants with time spent in Sweden due to *socioeconomic adversity*, *adaptation*, *racism* and *discrimination*). Ultimately, it is beyond our aim and study design to effectively disentangle and isolate these temporal factors.

For the second-generation in Fig. 5, a negative contribution in infancy grows stronger over time for boys and girls. This likely reflects the sizeable shift in composition of fertile-age migrant parents over time from Western (low infant mortality country origins) to non-Western (and high infant mortality country origins). Negative contributions in early to middle adulthood become stronger and pronounced over time. By the 2010s, the second-generation contribute systematically negatively to national PLE0 over age—albeit more strongly in the age ranges highlighted. The first-generation, meanwhile, show a complex pattern of contributing *positively* to national life expectancy in early to middle adulthood in Sweden and *negatively* at older adult ages.

**Fig. 6:**
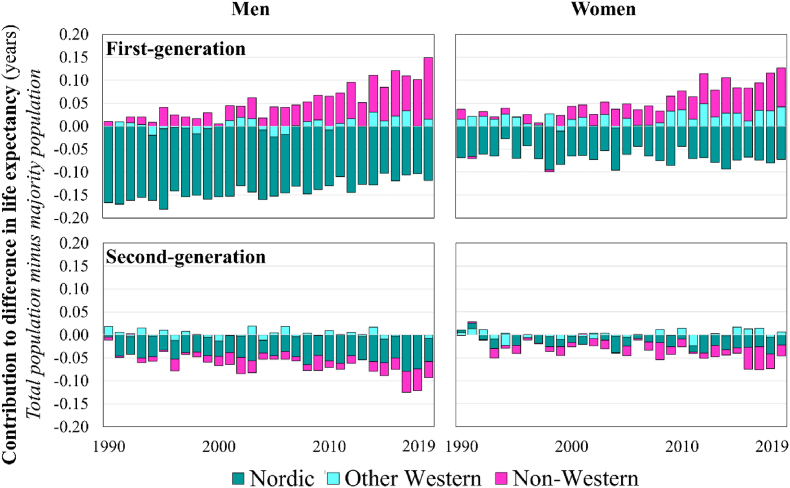
Generation by origin decompositions of life expectancy differences between total population and total population minus the first and second-generation, 1990–2019. Source: authors' calculations based upon Swedish register collection “Ageing Well”.

Fig. 6 displays the results of a decomposition in the difference in total PLE*0* and the PLE*0* of the majority population by generation and origin. First-generation Nordic migrants consistently contribute negatively to national PLE*0*, with impacts ranging from −0.10 years to −0.15 years for men and from −0.05 years to −0.10 years for women. Among first-generation men, the size of this negative effect is slowly decreasing. Conversely, first-generation non-Western migrants always contribute positively to national PLE*0*—even in instances when migrants, overall, do not, and this positive impact increases over time, reaching +0.09 years for women and +0.13 years for men by 2019. Other Western first-generation migrants make modest positive contributions to national PLE*0* throughout the period. For the second-generation, their overall negative impact is driven by people with at least one parent born in a Nordic country, with contributions ranging from −0.05 years to −0.09 years among men, and to a lesser extent, by those with at least one parent born in a non-Western country. The size of the negative effect of both origin groups is growing over time for men and women but is consistently smaller for women.

### 4.2 Supplementary analyses

We checked the accuracy of our estimates against Statistiska centralbyrån (SCB; the producer of official statistics in Sweden) and the Human Mortality Database (HMD; the world's leading scientific data resource on mortality in rich countries). The comparisons (in online Table 1) show a very high degree of consistency between our estimates and those of SCB and the HMD.

Table S2 shows the composition of first-generation Nordic, Western and non-Western groups according to detailed origins, and how they change over time and age. Due to the large amount of information provided, we only describe the composition at ‘all ages combined’. However, interested readers may want to refer to the age-specific tables when interpreting the age decompositions. Additionally, we note that these are *relative* shares (%)—an increase or decrease over time does not always equate to an increase or decrease in absolute numbers of migrants residing in Sweden. From 1990 to 2019, the composition of first-generation Nordic remains stable. Over 60% of migrants in this group are born in Finland. In 1990, the largest shares of other Western migrants are born in Central & Eastern Europe (37%) & Western Europe (39%). During the 1990s, we report rapidly rising shares of migrants born in former Yugoslavia (owing to the Balkans War), followed by growing shares of migrants from Central & Eastern Europe in the 2000s (owing to the accession of countries to the EU). In 2019, 41% of other Western migrants are born in Central & Eastern Europe, 28% in Western Europe, and 27% in former Yugoslavia. In 1990, most first-generation non-Western migrants are born in Central & Southern America (23%) and Iran & Iraq (21%), with smaller shares of many other groups (3–12%). Shares of Central & Southern America fall over time (to 8% by 2019). Shares of Iran & Iraq increase in the 2000s (up to 30%) and then decrease to 21% by 2019. We see gradually rising shares of migrants born in Sub-Saharan Africa (from 8% in 1990 to 17% by 2019), rapidly increasing shares of migrants born in Syria between 2013 (5%) and 2016 (17%), and slowly falling shares of Turks (12% in 1990 to 5% by 2019). Shares of all Asian groups are stable over time: Other Asians (5–7%), South East Asia (6–9%), South Asia (8–10%).

Table S3 Table S3 shows the composition of second-generation Nordic, Western and non-Western groups according to parental origins, and how they evolve over time and age. From 1990 to 2019, the composition of second-generation Nordic remains stable and comparable to the first-generation. Over 60% of second-generation in this group are born in Finland. In 1990, half of all other Western migrants are born in Western Europe (49%); this share falls over time to 36% by 2019. The share of second-generation Central & Eastern Europe, meanwhile, remains stable (at 28-30%). We see gradually rising shares of second-generation former Yugoslavia (up from 13% in 1990 to 26% in 2019). In 1990, most second-generation non-Western migrants are born in Central & Southern America and Turkey (both at 21%). Shares of Central & Southern America and Turkey fall over time (to 13% and 9% respectively by 2019). We see gradually rising shares of second-generation Sub-Saharan Africa (from 9% in 1990 to 18% by 2019) and Iran & Iraq (from 9% in 1990 to 19% by 2019) over time. Shares of all of the other groups are small and stable.

Expectedly, if you look within origins and ages and *across* Table S2 and Table S3, you can clearly see the age lag between parental age cohorts of migrants and the birth of their children.

Fig. S1 compares PLE*1* among first-generation Nordic, other Western, and non-Western migrants to the majority population. From 1990 to 2019, PLE*1* among migrant men and women from non-Western countries remains 2 to 3-years higher than that of majority population men and women. In contrast, PLE*1* is consistently 4–5 years lower among migrant men born in other Nordic countries and approximately 2-years lower among migrant women born in other Nordic countries than that of majority population men and women. The PLE*1* of migrant women born in other Western countries stays around half a year to a year higher than that of majority population women. Among migrant men born in other Western countries, PLE*1* oscillates around that of majority population men. The consistency of the difference in levels is remarkable, with no big temporal changes to report. We note that it was not possible to generate annual estimates for the second-generation groups due to their smaller population sizes and young age structure.

Figure S2 to Figure S5 present the results of decompositions by generation, age *and* origins. These figures contribute several extra insights *on top of* those provided by Fig. 5, Fig. 6 that we summarise here. First, the persistent negative contribution of first-generation men and women to national PLE*0* at older adult ages can be attributed predominantly to older first-generation migrants born in other Nordic countries. Second, the emergence of a positive contribution to national PLE*0* among first-generation men and women at young to middle adulthood between 1990 and 2019 is predominantly attributable to longevous migrants born in non-Western countries. Finally, the increasingly negative contribution of the second-generation to PLE*0* in infancy is attributable to high infant mortality among second-generation infants with non-Western origins.

Fig. S6 provides an additional decomposition. It examines the effect of having two foreign-born parents (middle column; G2.0) versus having one foreign-born and one native-born parent (right-hand column; G2.5) on the contributions of the second-generation to national PLE*0*. The figure decomposes the contribution of second-generation with *at least one* migrant parent from Fig. 4 (which is shown again in the left-hand column of Fig. S6). Both the G2.0 and the G2.5 contribute negatively to national PLE0. The size of the contribution is larger among the G2.0—a function of a larger number of deaths. However, the general patterns and trends are similar. Figure S6 (men) and Figure S7 (women) show age decompositions for the G2.0 and the G2.5. The age decomposition of the wider G2 (shown in Fig. 5) is provided for comparative purposes. From age 15-years, the patterns of the G2.0 and the G2.5 are very similar for men and women. They mirror the patterns previously described for the wider G2 in Fig. 5. It is in infancy and childhood that the age-specific contributions of the G2.0 and the G2.5 diverge. The G2.5 contribute *positively* to national PLE0 in infancy (particularly women). The G2.0, on the other hand, contribute increasingly *negatively* to national PLE0 in infancy and make consistent modest negative contributions to national PLE0 at childhood and adolescent ages. This difference likely reflects that the rates of intermarriage in Sweden are higher among non-migrants and migrants born in other parts of Europe (i.e., who come from low infant mortality origins) and substantially lower among non-migrants and migrants born in non-Western countries (i.e., who come from high infant mortality origins) (Dribe & Lundh, 2011)

## 5 Discussion

International migration has exerted profound and lasting demographic change in major host countries through the increasing share and diversification of the first-generation population *and* the establishment of the second-generation. One impact of this includes a growing potential for the first and second-generation to affect estimates of national mortality. In this article, in light of the unique mortality patterns experienced by migrants and the children of migrants (Aldridge et al., 2018; Shor & Roelfs, 2021; Wallace et al., 2023), we aimed to quantify and decompose the effect of the first-generation and second-generation on estimates of national life expectancy in Sweden. We placed specific emphasis on variation by generation, age, and origin (and combinations thereof), in light of a lack of evidence. We defined three questions, answered below.

**RQ1** asked, “*What is the average effect of the first and second-generation impact upon wider population health in Sweden in 1990? How does it change over time?*” For migrants, we found an initial negative effect on national life expectancy in 1990 that slowly reduced in size over and disappeared by 2019. In the context of the migrant mortality advantage (Aldridge et al., 2018; Shor & Roelfs, 2021) and evidence that migrants increasingly enhance national life expectancy in Australia (Page et al., 2007), the U.S., (Hendi & Ho, 2021; S. H. Preston & Elo, 2014), Denmark, Finland, and Norway (Wallace et al., 2022), Sweden goes firmly “against the grain”. Our finding is consistent with Wallace et al. (2022), who show similar levels and trends over time for Sweden. Unlike, Wallace et al., (2022), however, we move beyond a generational estimate in order to document substantial variation in the contributions of migrants to national PLE0 in Sweden by origins and age that we discuss in relation to our two remaining research questions.

Concerning the second-generation, our study follows only that of Hendi and Ho (2021) in quantifying the effect of the second-generation upon national mortality estimates. We found the opposite effect to Hendi and Ho (2021), who documented that the second-generation (when combined with the first-generation, an explicit estimate was not provided) further enhanced the positive effect of migrants on U.S. life expectancy. Here, we found a small initial negative effect in 1990 that developed into a larger negative effect by 2019. This contrast reflects the disparate mortality situations of second-generation in the U.S.—where a mortality advantage tends to be retained, at least in some residual form, in the second-generation—in comparison to the second-generation in Europe—where the mortality advantage of the first-generation tends to be lost and often reversed into a mortality disadvantage among the second-generation) (Wallace et al., 2023).

The combined first-generation and second-generation estimates mask the contrasting effects of migrants and the children of migrants on life expectancy in recent years. Namely, the emergence of a positive effect among migrants, alongside the continuation of an increasingly negatively impact among the children of migrants. This finding has implications for alternative measures of migrant background, such as race and ethnicity. Race and ethnicity both combine migrants and the children of migrants into one single group. Self-reporting of race and ethnicity has become more common in health research. Yet, country of birth still holds the advantage of objectivity and stability (Stronks et al., 2009). A reliance upon self-reported race and ethnicity produces race and ethnic groups whose composition varies by migrant status (i.e., born abroad versus born in the host country) and varies according to the length of a group's migration history. When the history of migration of a given group to the host country is *long*, a larger proportion of individuals belong to the second or higher generation. When it is short, a larger proportion of individuals belong to the first-generation (Khlat, 2023). The results of our study indicate that combining generations introduces unwanted heterogeneity that masks salient differences in life expectancy, and contributions to national life expectancy, according to first-generation and second-generation status. It may represent best practice to differentiate race and ethnic groups by generational status when studying health and mortality differences compared to the majority population in all countries with a long history of migration.

**RQ2** asked, “*At what ages do the first-generation and second-generation affect wider population health? How does this effect change over time?*” For migrants, the greatest effect– and transformation in effect–was concentrated in young to mid-adulthood (20-49-years). Over time, an initial, sizeable negative effect on national PLE0 at these ages reversed to become a positive effect on national PLE0 by 2019. Although there is a consistently negative influence of migrants on national PLE*0* at older adult ages over time, the peak of this negative effect has moved up the age range over time and compressed. This stark variation demonstrates the need to progress beyond a single estimate documenting the contribution of migrants to national mortality.

For example, during the most recent decade (2010-19), our analysis reveals that while the overall contribution of migrants to national life expectancy appears negligible, it conceals a complex age-specific impact. To elaborate, there are age ranges where migrants both elevate and depress national PLE0. Migrants contribute positively to national PLE0 in Sweden between ages 20–49 among men and ages 20–64 among women. Conversely, migrants depress national PLE0 between ages 50–89 among men and ages 65–89 among women. These contrasting effects effectively neutralize one another, resulting in a seemingly minimal overall impact of migrants during this time period. Yet, we must acknowledge these counteracting contributions when formulating conclusions about general progress in national mortality in Sweden. For example, in recent years Sweden has been “losing ground” in relation to other leading countries in life expectancy. This is because mortality at higher ages (65+) has improved more slowly than it has in other leading countries (Drefahl et al., 2014). In relation to our findings, although the contributions of the G1 at these older ages are unlikely to account fully for the broader patterns, Sweden's older – and predominantly Nordic – immigrant population must surely contribute to the country's underperformance in old age mortality relative to other countries. Among the G2, while the impact across different ages at least acts in the same direction over time, there are comparatively larger – and increasingly negative – effects on infancy (a crucial age in calculations of life expectancy) and early to mid-adult life.

**RQ3** asked, “*How do different immigrant origins affect national population health in Sweden? Does the influence of specific origins change over time?”* Sweden's case reveals the importance of the changing origin composition of a country's migrant population and a need to look beyond the generational level when assessing migration's role in wider population health. This is because one single origin group (G1 Nordic, the majority of which are of Finnish-origin) has played an era defining role in the generational patterns of migrant mortality advantage and disadvantage – and thus contribution to national mortality estimates – in the past few decades. It is too simplistic to state that immigrants have depressed population health in Sweden in most years. G1 non-Western migrants have always contributed positively to national PLE0 in Sweden and contribute increasingly positively over time. G2 Nordic and G2 non-Western children of migrants both negatively affect national PLE*0* – this negative effect is growing over time. Regarding other Western, the G1 mostly make a small positive contribution to national PLE0, while the impact of the G2 is sporadic.

There are many strengths to the study. First, we have used high quality Swedish register data and conducted analyses over a long period for millions of people, permitting the assessment of long-run patterns and trends. Second, we have provided new evidence concerning the influence of international migration on wider population health to a body of work (on the migrant mortality advantage) that has overwhelmingly treated migrant health as discrete and decontextualized from the health of the wider resident population of the host country. Third, we have investigated variation in this effect across origins, age, and sex, adding considerable nuance to a body of work that has largely provided a single average generational effect. Fourth, we adopted an intergenerational perspective, additionally quantifying the impact of the second-generation on wider population health – thus considering the wider impact of international migration. Potential weaknesses include an inability to investigate more granular origins (i.e., beyond Nordic, other Western and non-Western) and a lack of correction for death under-coverage and population over-coverage – errors that might serve to overestimate the effect of the first-generation on national population health. However, there is currently no agreed-upon method for correcting for this (potential) population under-coverage (Monti et al., 2019) and even less so for any (potential) death over-coverage.

With absolute and relative shares of first and second-generation projected to continue rising in Sweden (Karlsdottir et al., 2018) and in many other countries across Europe (Agafiţei & Ivan, 2016), the impact of international migration on mortality and life expectancy will only continue to grow. By contributing new empirical evidence concerning the effect of the first and second-generation on national mortality that includes substantial variation over generation, age and origins, we provide policy makers with a more nuanced understanding of how international migration affects measures used to track national progress in mortality. There is a case, given the unique, complex and divergent patterns and trends over generations, for the life expectancy of the majority population, first-generation, and second-generation to be monitored separately in to maximise national life expectancy gains in Sweden in the future. At the least, it seems clear that targeted public health policies aimed at reducing the elevated infant and early adult mortality risks of the second-generation and the excess older adult mortality of the first-generation would allow Sweden to generate renewed improvements in national life expectancy. We additionally recommend, in light of a recent large-scale review that reported elevated infant and early adult mortality among children of migrants in many European countries (that include Belgium, Denmark, France, the Netherlands, Norway, and the United Kingdom—countries where lower mortality risks among migrants have been consistently reported) (Wallace et al., 2023), that similar analyses be carried out across Europe to assess whether the life expectancy of the majority population, migrants, and children of migrants need to be monitored separately elsewhere.

## CRediT authorship contribution statement

**Matthew Wallace:** Writing – review & editing, Writing – original draft, Visualization, Validation, Software, Resources, Project administration, Methodology, Investigation, Funding acquisition, Formal analysis, Data curation, Conceptualization. **Sven Drefahl:** Writing – review & editing, Software, Methodology, Formal analysis, Conceptualization.

## Informed consent

Information from the administrative registers in Sweden is made available under official terms of secondary use. As such, people in the registers do not provide informed consent. Informed consent is not required for large-scale register-based studies in Sweden. It is instead assumed that the study participants do not object to research provided it is deemed ethical by an expert committee. Approval from an ethical committee (approval number below) therefore replaces individual approval in Sweden. Additionally, it is also assumed that the benefits that arise from register-based research far outweigh the potential risks regarding the new evidence that studies can generate, the policies that they can inform, and ultimately the positive change that they can effect.

## Ethical approval

Swedish Ethical Review Authority (dnr. 2017/1623-31/5)

## Data sharing

The data used for this study was accessed remotely through a secure data environment. Both Matthew Wallace and Sven Drefahl are ‘approved users’ of the data. ‘Approved users’ of this data tend to be individuals affiliated with a research institution that explicitly apply for access to the data by demonstrating considerable prior experience of handling sensitive, individual-level data. As such, we are unable to share the data. Those interested in using Swedish register data can see the website https://www.scb.se/en/services/ordering-data-and-statistics/ordering-microdata/mona--statistics-swedens-platform-for-access-to-microdata/, which provides links and information on how to officially access Swedish register data, including a range of meta data.

## Funding

The work was funded through two grants from the 10.13039/501100006636Swedish Research Council for Health, Working Life and Welfare (10.13039/501100006636FORTE) grant 2019-00603 ‘Migrant mortality advantage lost? Emerging lifespan inequalities among migrants and their descendants in Sweden’ and grant 2016–07115 ‘Ageing Well’, and the U.K. Research and Innovation (10.13039/100014013UKRI) grant Horizon Europe Guarantee grant APP44827 ‘Living longer in poorer health? Understanding the immigrant morbidity-mortality paradox’.

## Declaration of interest statement

Matthew Wallace and Sven Drefahl have nothing to declare.

## Data Availability

The authors do not have permission to share data.

## Footnotes

1 2014 represents the most recent year with data available on the absolute and relative shares of first- and second-generation living in a European Union country.

2 Our own more recent calculations based upon the Swedish register data collection “Ageing Well” that we use for the analysis place this relative share closer to 33% in 2019—the final year of our analysis.

## References

[bib1] Acevedo-Garcia D., Sanchez-Vaznaugh E.V., Viruell-Fuentes E.A., Almeida J. (2012). Integrating social epidemiology into immigrant health research: A cross-national framework. Social Science & Medicine.

[bib2] Agafiţei M., Ivan G. (2016). First and second-generation immigrants—statistics on main characteristics. https://ec.europa.eu/eurostat/statistics-explained/index.php?title=First_and_second-generation_immigrants_-_statistics_on_main_characteristics.

[bib3] Aldridge R.W., Nellums L.B., Bartlett S., Barr A.L., Patel P., Burns R., Hargreaves S., Miranda J.J., Tollman S., Friedland J.S., Abubakar I. (2018). Global patterns of mortality in international migrants: A systematic review and meta-analysis. The Lancet.

[bib4] Auger N., Feuillet P., Martel S., Lo E., Barry A.D., Harper S. (2014). Mortality inequality in populations with equal life expectancy: Arriaga's decomposition method in SAS, Stata, and Excel. Annals of Epidemiology.

[bib5] Batalova J.B., C E., J (2022). https://www.migrationpolicy.org/article/frequently-requested-statistics-immigrants-and-immigration-united-states.

[bib6] Bevelander P., Bilder R., Dahlstedt I., Eskelund M., Hansen L., Macura M., Pedersen K., Ostby L. (2013).

[bib7] Borevi K. (2014). Multiculturalism and welfare state integration: Swedish model path dependency. Identities: Global Studies in Power and Culture.

[bib8] Chiang C. (1984).

[bib9] Coleman D. (2008). The demographic effects of international migration in Europe. Oxford Review of Economic Policy.

[bib10] Drefahl S., Ahlbom A., Modig K. (2014). Losing ground—Swedish life expectancy in a comparative perspective. PLoS One.

[bib41] Dribe M., Lundh C. (2011). Cultural dissimilarity and intermarriage. A longitudinal study of immigrants in Sweden 1990–2005 1. International migration review.

[bib11] Guillot M., Khlat M., Elo I., Solignac M., Wallace M. (2018). Understanding age variations in the migrant mortality advantage: An international comparative perspective. PLoS One.

[bib12] Hedberg C., Kepsu K. (2003). Migration as a cultural expression? The case of the Finland-Swedish minority's migration to Sweden. Geografiska Annaler - Series B: Human Geography.

[bib13] Hendi A.S., Ho J.Y. (2021). Immigration and improvements in American life expectancy. SSM - Population Health.

[bib14] Hiam L., Zhang C.X., Burns R., Darlington-Pollock F., Wallace M., McKee M. (2022). What can the UK learn from the impact of migrant populations on national life expectancy?. Journal of Public Health, Accepted, forthcoming.

[bib15] Ho J.Y., Hendi A.S. (2018). Recent trends in life expectancy across high income countries: Retrospective observational study. BMJ.

[bib16] Hyndman R.J., Booth H., Tickle L., Maindonald J. (2019).

[bib17] Karlsdottir A., Rispling L., Norlen G., Randall L., Gassen N.S., Heleniak T., Peurell E., Rehn-Mendoza N., Lagercrantz H. (2018).

[bib18] Keyfitz N., Caswell H. (2005).

[bib19] Khlat M. (2023). Revisiting the disproportionate COVID-19 mortality of ethnic minorities in light of the migrant mortality advantage. Frontiers in Public Health.

[bib20] Kobori E., Maeda Y., Yamamoto Y. (2017). [Mortality rates of foreign national residents in Japan: Comparison with the Japanese population and a possible healthy migrant effect]. [Nihon Koshu Eisei Zasshi] Japanese Journal of Public Health.

[bib21] Korkiasaari J., Söderling I. (2003). Finnish emigration and immigration after world war II. Siirtolaisuusinstituutti – Migrationsinstitutet.

[bib22] Luy M., Di Giulio P., Di Lego V., Lazarevič P., Sauerberg M. (2020). Life expectancy: Frequently used, but hardly understood. Gerontology.

[bib23] Maret-Ouda J., Tao W., Wahlin K., Lagergren J. (2017). Nordic registry-based cohort studies: Possibilities and pitfalls when combining Nordic registry data. Scandinavian Journal of Public Health.

[bib24] Mehta N.K., Elo I.T., Engelman M., Lauderdale D.S., Kestenbaum B.M. (2016). Life expectancy among U.S.-born and foreign-born older adults in the United States: Estimates from linked social security and medicare data. Demography.

[bib25] Migrationsverket (2020). *Migration to Sweden* [text]. https://www.migrationsverket.se/English/About-the-Migration-Agency/Migration-to-Sweden/History.html.

[bib26] Monti A., Drefahl S., Mussino E., Härkönen J. (2019). Over-coverage in population registers leads to bias in demographic estimates. Population Studies.

[bib27] Page A., Begg S., Taylor R., Lopez A.D. (2007). Global comparative assessments of life expectancy: The impact of migration with reference to Australia. Bulletin of the World Health Organization.

[bib28] Pedersen P.J., Røed M., Wadensjö E. (2008).

[bib29] Preston S.H., Elo I.T. (2014). Anatomy of a municipal triumph: New York city's upsurge in life expectancy. Population and Development Review.

[bib30] Preston S., Heuveline P., Guillot M. (2001).

[bib31] Schierup C.-U., Ålund A. (2011). The end of Swedish exceptionalism? Citizenship, neoliberalism and the politics of exclusion. Race & Class.

[bib32] Shor E., Roelfs D. (2021). A global meta-analysis of the immigrant mortality advantage. International Migration Review.

[bib33] Statistiska Centralbyrån (2021). Life expectancy 1751–2021. Statistiska Centralbyrån.

[bib34] Stronks K., Kulu-Glasgow I., Agyemang C. (2009). The utility of ‘country of birth’ for the classification of ethnic groups in health research: The Dutch experience. Ethnicity and Health.

[bib35] Trovato F., Odynak D. (2011). Sex differences in life expectancy in Canada: Immigrant and native-born populations. Journal of Biosocial Science.

[bib36] United Nations Department of Economic and Social Affairs (2019).

[bib37] Wallace M. (2023). Handbook on migration and ageing.

[bib38] Wallace M., Hiam L., Aldridge R.W. (2023). Elevated mortality among second-generation (children of migrants): What is going wrong in Europe?. British Medical Bulletin.

[bib39] Wallace M., Thomas M.J., Aburto J.M., Jørring Pallesen A.V., Mortensen L.H., Syse A., Drefahl S. (2022). Immigration, mortality, and national life expectancy in the Nordic region, 1990–2019. SSM - Population Health.

[bib40] Wallace M., Wilson B. (2022). Age variations and population over-coverage: Is low mortality among migrants merely a data artefact?. Population Studies.

