# Supplementary material for: Against the grain: International migrants, the children of migrants and national life expectancy in Sweden, 1990–2019
Source: SSM Popul Health. 2024 Nov 8;28:101726. doi: 10.1016/j.ssmph.2024.101726 (PMC11582771; doi:10.1016/j.ssmph.2024.101726)
Supplement: Multimedia component 1 [file mmc1.pdf]

Box S1. Country of birth composition of Nordic, other Western (non-Nordic), and non-Western

<table>
<tr><td rowspan="4">Nordic</td><td colspan="2">Denmark</td></tr>
<tr><td colspan="2">Finland</td></tr>
<tr><td colspan="2">Iceland</td></tr>
<tr><td colspan="2">Norway</td></tr>
<tr><td rowspan="24">Western</td><td rowspan="2">Former Yugoslavia</td><td>Bosnia & Herzegovina</td></tr>
<tr><td>Former Yugoslavia</td></tr>
<tr><td rowspan="7">Other Central & Eastern Europe</td><td>Bulgaria</td></tr>
<tr><td>Czech Republic</td></tr>
<tr><td>Hungary</td></tr>
<tr><td>Poland</td></tr>
<tr><td>Romania</td></tr>
<tr><td>Former Soviet Union</td></tr>
<tr><td>Other Central & Eastern Europe</td></tr>
<tr><td rowspan="11">Western Europe</td><td>Austria</td></tr>
<tr><td>Belgium</td></tr>
<tr><td>British Isles</td></tr>
<tr><td>France</td></tr>
<tr><td>Germany</td></tr>
<tr><td>Greece & Cyprus</td></tr>
<tr><td>Italy</td></tr>
<tr><td>The Netherlands</td></tr>
<tr><td>Portugal</td></tr>
<tr><td>Spain</td></tr>
<tr><td>Switzerland</td></tr>
<tr><td rowspan="4">USA-Canada-Australia-NZ</td><td>Australia</td></tr>
<tr><td>Canada</td></tr>
<tr><td>New Zealand</td></tr>
<tr><td>United States</td></tr>
<tr><td rowspan="23">Non-Western</td><td rowspan="3">Central & Southern America</td><td>Chile</td></tr>
<tr><td>Other Central & Southern America</td></tr>
<tr><td>Central America & the Caribbean</td></tr>
<tr><td rowspan="4">Sub-Saharan Africa</td><td>Ethiopia</td></tr>
<tr><td>Eritrea</td></tr>
<tr><td>Somalia</td></tr>
<tr><td>Other Sub-Saharan Africa</td></tr>
<tr><td rowspan="7">Middle East, Northern Africa and Turkey (MENAT)</td><td>Lebanon</td></tr>
<tr><td>Iran</td></tr>
<tr><td>Iraq</td></tr>
<tr><td>Syria</td></tr>
<tr><td>Turkey</td></tr>
<tr><td>Northern Africa</td></tr>
<tr><td>Other Arab countries</td></tr>
<tr><td rowspan="4">Southern Asia</td><td>Afghanistan</td></tr>
<tr><td>Bangladesh & Pakistan</td></tr>
<tr><td>India</td></tr>
<tr><td>Other South Asia</td></tr>
<tr><td rowspan="3">South-East Asia</td><td>Philippines</td></tr>
<tr><td>Thailand</td></tr>
<tr><td>Vietnam</td></tr>
<tr><td rowspan="2">Other Asia</td><td>China</td></tr>
<tr><td>Other Asia</td></tr>
<tr><td>Rest of the World</td><td>Rest of the World</td><td>Rest of the World</td></tr>
</table>

Notes: the level of detail represents the lowest level of detail that we have available to us in our data. All the labels are consistent with the labels provided to us by the data provider.

Source: author's calculations based upon Swedish register collection "Ageing Well".

Box S2. Life expectancy at birth or age one?

Evidence exists that calculating life expectancy at birth among migrants may artificially inflate the value of this health metric. This is for two interrelated reasons: 1) Infant mortality represents an important and influential age in the calculations of the demographic life table; 2) So few migrants arrive in the first year of life (and particularly in the first 28-days, where the risk of death is highest among infants) that often there are zero deaths at this age in national migrant population data. Consequently, this leads to a partial replication of the first row of the life table (age 0) in the second row (at age 1). This adds a full year to migrants' life expectancy at birth. Infant deaths *are* occurring in migrants' countries of origin, but these children are simply not crossing borders. Estimating in life expectancy at age one is more technically correct in for the lifetable. However, it does potentially ignore an important aspect of immigration—**selection**. Namely, parents do not move with infants and even more so, parents do not move with sick infants. There is some argument to be made that life expectancy at age one ignores this.

This logic has been used by Ho and Hendi (2021), Wallace et al. (2022) to avoid using life expectancy at birth in calculations of migrant life expectancy. Wallace et al. (2022) have conducted a sensitivity analysis regarding this problem. It can be found in Table S7 and Table S8 of the online materials in their *Social Science & Medicine Population Health*. It shows two things.

First, that the size of the gap in life expectancy at birth between migrants and non-migrants *is* larger than the gap in life expectancy at age one between migrants and non-migrants. **In the main body of the paper, we do not include explicit estimates of migrants' life expectancy (at age 0, 1, or otherwise).** We do, however, include a life expectancy at birth estimate for a combined first-generation and second-generation. Nevertheless, the logic behind estimating life expectancy at age one for this particular population is far less clear cut than it would be for migrants alone. The second-generation are born in Sweden and **exposed to the risk of infant mortality from day one**. This population is responsible for a sizeable share of annual births in Sweden (starting at 18% of births to at least one foreign-born parent in 1990 and rising to 37% in 2019) and deaths (starting at 19% infant deaths to at least one foreign-born parent in 1990 and rising to 46% by 2019). We also know that the second-generation has a higher risk of infant mortality in Sweden (6.34 [G2] versus 6.20 [non-migrant] deaths per

1,000 live births in 1990 to 2.57 [G2] versus 1.76 [non-migrant] deaths per 1,000 live births in 2019)[1].

What would be the justification for life expectancy at age one for this combined group? While one might be able to justify removing age 0 for this combined estimate to counteract the small amount of bias induced in life expectancy at birth estimates by a lack of 'migrant' infant mortality, it would make little sense to remove age 0 for the second-generation (something that would have a considerably larger impact). Given that the aim of the paper is to assess the impact of the mortality of migrants and their children on national mortality in Sweden, we definitely want to capture and estimate **the importance of these early life survival inequalities** of the second-generation upon national mortality patterns and trends in Sweden. Consequently, we believe that there is a much more compelling case to use life expectancy at birth so as to capture a key age at which the second-generation in Sweden has its most tangible effect on national mortality.

Second, this issue, precisely due to a lack of deaths and exposure at age 0— **does not lead to an over-estimation of the gap in life expectancy at birth between the total resident population and majority population** (i.e., the metric that we use here to measure the impact of the first generation and second-generation upon national mortality patterns and trends in Sweden). Indeed, Wallace et al. (2022) find absolutely no difference (to two decimal places) in the gap between life expectancy at birth between the total and majority population (i.e., total population minus first-generation) and life expectancy at age 1 between the total and majority population.

We note that in the supplementary materials, where we do provide estimates of migrant-specific life expectancy according to their regions of birth, we do so using life expectancy at age 1.

[1] Author's calculations based upon register collection "Ageing Well"

Box S3. Arriaga decomposition method

${}_{n}\triangle_{x}$ shows the contribution to the difference in life expectancy between population *1* and *2* from all-cause mortality in age group *x* to *x* + *n* based upon life table functions l_x, T_x , and ${}_{n}L_{x}$.

$$(1)\ {}_{n}\triangle_{x} = \frac{l_x^1}{l_o^1} \cdot \left(\frac{{}_{n}L_x^2}{l_x^2} - \frac{{}_{n}L_x^1}{l_x^1} \right) + \frac{T_{x+n}^2}{l_0^1} \cdot \left(\frac{l_x^1}{l_x^2} - \frac{l_{x+n}^1}{l_{x+n}^2} \right)$$

We expand the decomposition with:

$$(2)\ {}_{n}\triangle_{x}^{i} = {}_{n}\triangle_{x} \cdot \frac{{}_{n}R_x^i(2) \cdot {}_{n}m_x(2) - {}_{n}R_x^i(1) \cdot {}_{n}m_x(1)}{{}_{n}m_x(2) - {}_{n}m_x(1)}$$

Where the ${}_{n}\triangle_{x}^{i}$ is the contribution to the difference in life expectancy for individual or parental origin i in age group x to x + n, and the contribution that specific individual or parental origins gives to the difference in life expectancy between the two populations. ${}_{n}m_{x}(1)$ and ${}_{n}m_{x}(2)$ represents the death rate for age group *x* to *x* + *n* and ${}_{n}R_x^i(1)$ and ${}_{n}R_x^i(2)$ is the share of deaths from origin *i* for age group *x* to *x* + *n* in the two populations. As described in the equation above, ${}_{n}\triangle_{x}$ represents the difference in mortality from all origins in age group *x* to *x* + *n*.

Table S1. Comparison of our national life expectancy at birth estimates to the Human Mortality Database and Statistiska centralbyrån between 1990 and 2019 (Statistics Sweden; SCB)

Year	Men					Women				
	Our estimate	Human Mortality Database	Difference	Statistiska centralbyrån (SCB)	Difference	Our estimate	Human Mortality Database	Difference	Statistiska centralbyrån (SCB)	Difference
1990	74.79	74.81	-0.02	74.81	-0.02	80.39	80.40	-0.01	80.41	-0.02
1991	74.93	74.95	-0.02	74.94	-0.01	80.54	80.54	0.00	80.54	0.00
1992	75.35	75.36	-0.01	75.35	0.00	80.78	80.78	0.00	80.79	-0.01
1993	75.47	75.49	-0.02	75.49	-0.02	80.76	80.78	-0.02	80.79	-0.03
1994	76.06	76.08	-0.02	76.08	-0.02	81.38	81.38	0.00	81.38	0.00
1995	76.17	76.18	-0.01	76.17	0.00	81.45	81.44	0.01	81.45	0.00
1996	76.51	76.52	-0.01	76.51	0.00	81.52	81.52	0.00	81.53	-0.01
1997	76.69	76.70	-0.01	76.70	-0.01	81.79	81.80	-0.01	81.82	-0.03
1998	76.81	76.87	-0.06	76.87	-0.06	81.89	81.91	-0.02	81.94	-0.05
1999	76.99	77.07	-0.08	77.06	-0.07	81.86	81.89	-0.03	81.91	-0.05
2000	77.34	77.38	-0.04	77.38	-0.04	82.01	82.02	-0.01	82.03	-0.02
2001	77.51	77.54	-0.03	77.55	-0.04	82.05	82.05	0.00	82.07	-0.02
2002	77.69	77.71	-0.02	77.73	-0.04	82.08	82.08	0.00	82.11	-0.03
2003	77.88	77.91	-0.03	77.91	-0.03	82.41	82.41	0.00	82.43	-0.02
2004	78.33	78.35	-0.02	78.35	-0.02	82.64	82.66	-0.02	82.68	-0.04
2005	78.39	78.42	-0.03	78.43	-0.04	82.75	82.75	0.00	82.77	-0.02
2006	78.66	78.69	-0.03	78.70	-0.04	82.89	82.90	-0.01	82.91	-0.02
2007	78.90	78.93	-0.03	78.92	-0.02	82.93	82.94	-0.01	82.95	-0.02
2008	79.06	79.09	-0.03	79.09	-0.03	83.10	83.12	-0.02	83.13	-0.03
2009	79.30	79.34	-0.04	79.33	-0.03	83.32	83.33	-0.01	83.33	-0.01
2010	79.48	79.52	-0.04	79.52	-0.04	83.46	83.47	-0.01	83.49	-0.03
2011	79.77	79.80	-0.03	79.79	-0.02	83.65	83.67	-0.02	83.67	-0.02
2012	79.84	79.87	-0.03	79.87	-0.03	83.52	83.53	-0.01	83.54	-0.02
2013	80.05	80.10	-0.05	80.09	-0.04	83.70	83.72	-0.02	83.71	-0.01
2014	80.31	80.35	-0.04	80.35	-0.04	84.04	84.05	-0.01	84.05	-0.01
2015	80.29	80.32	-0.03	80.31	-0.02	84.00	84.02	-0.02	84.01	-0.01
2016	80.53	80.57	-0.04	80.56	-0.03	84.07	84.08	-0.01	84.09	-0.02
2017	80.69	80.73	-0.04	80.72	-0.03	84.10	84.12	-0.02	84.10	0.00
2018	80.77	80.79	-0.02	80.78	-0.01	84.24	84.26	-0.02	84.25	-0.01
2019	81.35	81.35	0.00	81.34	0.01	84.74	84.73	0.01	84.73	0.01

Source: author's calculations based upon Swedish register collection "Ageing Well"

Table S2. The changing age-origin composition of G1 Nordic, Western and non-Western, 1990-2019.

**First-generation** *(all ages)*						1990s										2000s										2010s					
	89	90	91	92	93	94	95	96	97	98	99	00	01	02	03	04	05	06	07	08	09	10	11	12	13	14	15	16	17	18	19
	%	%	%	%	%	%	%	%	%	%	%	%	%	%	%	%	%	%	%	%	%	%	%	%	%	%	%	%	%	%	%
**Nordic**																															
Finland	69	68	69	69	69	70	70	70	70	70	70	70	69	68	68	67	67	66	65	65	65	64	64	64	64	64	63	63	63	63	64
Denmark	14	14	14	14	14	14	14	14	14	14	14	14	14	14	15	15	16	16	17	17	17	17	17	17	17	17	17	17	17	17	17
Iceland	1	1	1	1	1	1	1	1	1	1	1	1	1	1	1	1	1	1	1	2	2	2	2	2	2	2	2	2	2	2	2
Norway	16	17	16	16	16	15	15	15	15	15	15	15	16	16	16	16	16	16	16	16	16	17	17	17	17	17	17	17	18	17	17
**Other Western**																															
Former Yugoslavia	17	17	18	18	25	34	36	36	36	37	36	37	36	36	36	36	36	35	34	33	32	31	30	30	29	28	28	28	27	28	28
Central & Eastern Europe	37	38	38	38	35	31	31	31	31	30	30	30	30	30	30	31	32	33	34	35	36	37	38	39	39	40	40	40	41	41	41
Western Europe	39	38	37	37	33	29	28	28	28	28	28	28	28	28	28	28	27	27	27	27	27	27	27	27	27	27	27	27	27	27	27
USA, Can, Aus, NZ	7	7	7	7	6	5	5	5	5	5	5	5	6	6	6	5	5	5	5	5	5	5	5	5	5	5	5	5	5	5	5
**Non-Western**																															
Central & Southern America	23	22	21	20	19	18	18	18	17	17	17	16	16	16	15	15	15	14	13	13	12	12	12	11	11	10	9	9	8	8	8
SS Africa	8	9	10	11	11	12	12	12	12	12	12	12	12	12	12	12	12	13	13	13	14	15	16	16	17	18	18	18	17	17	17
Other Middle East	10	11	11	11	11	11	11	11	10	10	10	10	10	9	9	9	9	9	9	9	9	9	9	9	9	8	8	8	8	8	8
Syria	3	3	4	4	4	4	4	4	3	4	4	4	4	4	3	3	3	3	3	3	3	3	3	4	5	8	11	16	17	17	17
Iran & Iraq	21	21	22	22	23	23	24	24	25	26	26	27	28	29	29	28	28	28	29	30	30	29	28	28	26	25	23	21	21	21	21
Turkey	12	11	11	10	10	10	10	9	9	9	9	9	8	8	8	8	8	8	7	7	7	7	7	6	6	6	5	5	5	5	5
Other Asia	7	7	6	6	6	6	6	6	6	6	6	6	6	6	6	6	6	6	6	6	6	6	6	6	6	6	5	5	5	5	5
South East Asia	6	6	6	7	7	7	7	7	7	7	7	7	7	8	8	8	8	9	9	9	9	9	9	9	9	8	8	7	7	7	7
South Asia	10	9	9	9	9	8	8	9	9	9	9	9	9	9	9	9	9	9	9	9	10	10	10	10	10	10	10	10	11	11	11
Rest of the World	1	1	1	1	1	1	1	1	1	1	1	1	1	1	1	1	1	1	1	1	1	1	1	1	1	1	1	1	1	1	1

**First-generation** *(0-14 years-old)*						1990s										2000s										2010s					
	89	90	91	92	93	94	95	96	97	98	99	00	01	02	03	04	05	06	07	08	09	10	11	12	13	14	15	16	17	18	19
	%	%	%	%	%	%	%	%	%	%	%	%	%	%	%	%	%	%	%	%	%	%	%	%	%	%	%	%	%	%	%
**Nordic**																															
Finland	37	31	29	29	30	31	32	32	34	35	35	34	32	30	28	26	24	22	20	19	17	17	16	15	15	15	16	16	16	16	16
Denmark	21	22	22	23	22	22	22	22	22	22	22	22	23	24	25	27	30	34	37	38	39	39	38	37	37	35	34	32	30	30	29
Iceland	10	12	12	12	12	12	13	11	10	9	8	7	6	6	6	6	6	5	5	6	7	7	8	9	9	9	10	10	9	9	9
Norway	31	35	36	36	36	34	34	34	34	34	35	37	39	41	42	41	40	39	38	37	37	37	37	38	40	41	41	42	44	45	47
**Other Western**																															
Former Yugoslavia	9	11	13	14	41	63	63	61	60	58	56	53	49	45	40	34	27	21	15	13	12	10	9	9	8	8	8	8	9	9	9
Central & Eastern Europe	49	50	51	51	35	22	22	22	21	22	22	22	23	24	25	28	31	36	39	40	42	43	44	44	44	43	43	41	40	40	39
Western Europe	31	28	26	25	17	11	11	12	13	14	16	18	20	22	25	28	31	33	35	36	36	36	36	37	37	38	38	40	40	41	41
USA, Can, Aus, NZ	11	11	10	10	7	5	5	5	6	6	7	7	8	9	9	10	11	11	11	10	11	11	11	11	11	11	11	10	11	11	11
**Non-Western**																															
Central & Southern America	28	26	24	23	21	20	19	19	18	17	16	16	15	14	13	12	12	11	10	9	8	7	7	7	6	5	4	3	3	3	3
SS Africa	5	6	7	9	10	12	12	13	13	13	13	12	12	11	11	12	12	13	15	17	20	22	23	24	29	28	25	22	20	19	19
Other Middle East	8	9	10	10	10	10	10	9	9	8	8	7	6	6	5	5	6	6	6	6	5	6	6	6	6	7	7	8	8	8	8
Syria	2	2	3	3	3	3	3	3	2	2	2	2	2	1	1	1	1	2	1	1	1	2	2	4	9	17	25	34	36	38	39
Iran & Iraq	18	19	20	21	22	23	23	23	24	26	28	30	31	33	34	32	29	28	30	32	32	29	27	24	19	16	13	11	10	10	9
Turkey	5	5	5	4	4	4	4	4	4	4	3	3	3	3	2	2	2	2	2	2	2	2	2	2	2	2	2	2	2	2	2
Other Asia	9	8	8	8	8	8	8	8	8	9	9	10	10	10	11	12	13	13	13	12	12	12	12	12	10	9	8	7	6	5	5
South East Asia	6	6	5	6	6	6	7	7	8	8	9	10	10	11	12	13	14	14	13	13	12	12	12	11	9	8	7	6	5	5	5
South Asia	19	18	17	16	15	14	14	13	13	12	11	11	11	11	10	10	10	9	8	8	7	7	8	9	9	9	8	8	9	9	9
Rest of the World	0	0	0	0	0	0	0	0	0	0	0	0	0	1	1	1	1	1	1	1	1	1	1	1	1	1	1	1	1	1	1

**First-generation** *(15-39 years-old)*						1990s										2000s										2010s					
	89	90	91	92	93	94	95	96	97	98	99	00	01	02	03	04	05	06	07	08	09	10	11	12	13	14	15	16	17	18	19
	%	%	%	%	%	%	%	%	%	%	%	%	%	%	%	%	%	%	%	%	%	%	%	%	%	%	%	%	%	%	%
**Nordic**																															
Finland	72	69	69	69	69	68	68	67	67	66	65	63	60	57	54	51	49	46	43	40	38	38	38	37	38	38	37	38	38	37	36
Denmark	12	12	12	12	12	13	13	13	13	14	15	15	17	19	20	22	24	27	30	31	32	32	31	31	30	28	28	27	27	27	27
Iceland	2	3	3	3	3	3	3	3	3	3	3	3	3	3	3	3	4	4	4	4	5	5	6	6	6	7	7	7	7	7	7
Norway	14	17	17	16	16	16	16	16	17	17	18	19	20	22	22	23	23	24	24	25	25	25	25	26	26	27	28	28	29	29	30
**Other Western**																															
Former Yugoslavia	21	21	21	20	30	42	43	43	44	44	44	44	43	43	43	43	42	41	39	36	34	33	31	30	29	28	27	26	25	25	26
Central & Eastern Europe	34	34	35	36	32	27	27	26	26	26	26	26	27	27	28	29	30	33	36	38	40	41	42	43	44	44	45	45	45	45	45
Western Europe	36	36	35	34	29	24	24	24	23	23	23	23	23	23	23	22	21	21	21	21	21	21	21	22	22	23	23	24	25	25	24
USA, Can, Aus, NZ	9	9	9	10	9	7	7	7	7	7	7	7	7	7	7	6	6	5	5	5	5	5	5	5	5	5	5	5	5	5	5
**Non-Western**																															
Central & Southern America	20	19	18	17	17	16	16	16	16	16	15	15	15	15	15	14	14	13	13	12	12	11	11	10	10	9	8	7	7	6	6
SS Africa	10	11	12	12	13	13	14	14	14	14	14	14	14	14	14	14	14	14	14	14	15	16	17	17	18	19	20	20	20	20	21
Other Middle East	10	11	11	11	11	11	11	11	11	11	10	10	10	10	10	9	9	9	9	9	8	8	8	8	8	8	8	8	7	7	7
Syria	3	3	4	4	4	4	4	4	4	4	4	4	4	4	4	3	3	3	3	3	3	3	3	3	5	8	11	16	17	17	18
Iran & Iraq	24	24	23	23	24	24	24	24	24	25	25	25	26	26	26	26	26	27	28	28	28	27	27	26	25	23	22	20	19	19	19
Turkey	13	12	11	11	10	10	10	9	9	9	9	8	8	8	7	7	7	7	6	6	6	6	6	5	5	5	4	4	3	3	3
Other Asia	6	6	6	6	6	6	6	6	6	6	6	6	6	6	6	6	6	6	6	6	7	7	7	6	6	6	5	5	5	5	5
South East Asia	7	7	7	8	8	8	8	8	8	8	8	8	8	8	8	8	9	9	9	9	9	9	9	9	9	8	8	7	6	6	6
South Asia	7	7	7	7	7	7	8	8	8	9	9	9	9	10	10	11	11	11	11	12	12	13	13	13	14	13	13	13	14	14	14
Rest of the World	1	1	1	1	1	1	1	1	1	1	0	0	1	1	1	1	1	1	1	1	1	1	1	1	1	1	1	1	1	1	1

Notes: percentages are column percentages *within* the three broader parental origin groups.

Source: author's calculations based upon Swedish register collection "Ageing Well"

Table S2 (cont.) The changing age-origin composition of G1 Nordic, Western and non-Western, 1990-2019.

**First-generation** *(40-64 years-old)*						1990s										2000s										2010s					
	89 %	90 %	91 %	92 %	93 %	94 %	95 %	96 %	97 %	98 %	99 %	00 %	01 %	02 %	03 %	04 %	05 %	06 %	07 %	08 %	09 %	10 %	11 %	12 %	13 %	14 %	15 %	16 %	17 %	18 %	19 %
**Nordic**																															
Finland	74	74	75	76	77	77	77	78	78	78	78	78	78	77	76	76	75	74	73	73	72	72	71	70	69	67	66	66	65	64	63
Denmark	13	13	13	12	12	12	12	11	11	11	11	11	11	11	11	12	12	12	13	13	13	14	14	15	15	16	16	16	17	17	17
Iceland	0	1	1	1	1	1	1	1	1	1	1	1	1	1	1	1	1	1	1	1	1	1	2	2	2	2	2	2	2	2	3
Norway	12	12	11	11	11	11	10	10	10	10	10	10	11	11	11	12	12	12	13	13	13	13	14	14	14	15	15	16	16	17	17
**Other Western**																															
Former Yugoslavia	19	20	20	20	23	29	31	32	32	33	34	34	35	36	36	37	37	37	37	37	36	36	36	36	35	34	34	33	32	32	32
Central & Eastern Europe	33	33	34	34	34	32	32	32	32	32	32	32	32	31	31	31	31	32	32	33	34	35	35	35	36	37	38	39	39	40	40
Western Europe	44	43	42	42	39	36	34	33	32	32	31	30	29	29	28	28	27	27	26	26	25	25	24	24	24	24	24	24	23	23	23
USA, Can, Aus, NZ	4	4	4	4	4	3	3	3	3	3	4	4	4	4	4	4	4	4	4	4	5	5	5	5	5	5	5	5	5	5	5
**Non-Western**																															
Central & Southern America	27	25	25	24	23	22	21	21	20	20	19	18	18	17	17	17	16	16	15	15	14	14	13	13	13	12	12	11	11	11	11
SS Africa	7	8	8	8	8	9	9	9	9	9	9	10	10	10	10	10	10	11	11	11	12	13	13	13	13	14	14	14	14	14	15
Other Middle East	11	11	12	12	11	11	11	11	11	10	10	10	10	10	10	10	10	10	10	10	10	10	10	10	10	10	9	9	9	9	9
Syria	4	4	4	4	4	4	4	4	4	4	4	4	4	4	4	4	4	4	4	4	4	4	4	4	5	7	9	11	12	12	12
Iran & Iraq	16	18	20	21	22	23	24	25	26	28	29	30	30	31	31	31	31	31	32	32	32	31	31	30	29	28	27	26	25	25	24
Turkey	15	14	13	13	12	12	12	12	11	11	10	10	10	10	10	9	9	9	9	9	8	8	8	8	8	8	7	7	7	6	6
Other Asia	7	7	6	6	6	5	5	5	5	4	4	4	4	4	4	4	4	4	4	4	4	4	5	5	5	5	5	5	4	4	4
South East Asia	5	5	5	5	5	6	6	6	6	6	6	6	7	7	7	7	7	8	8	8	8	9	9	9	9	9	9	9	9	9	9
South Asia	7	7	7	7	7	7	7	7	7	7	7	7	7	7	7	7	7	7	7	7	7	7	7	7	7	7	7	7	8	8	8
Rest of the World	1	1	1	1	1	1	1	1	1	1	1	1	1	1	1	1	1	1	1	1	1	1	1	1	1	1	1	1	1	1	1

**First-generation** *(65 years-old plus)*						1990s										2000s										2010s					
	89 %	90 %	91 %	92 %	93 %	94 %	95 %	96 %	97 %	98 %	99 %	00 %	01 %	02 %	03 %	04 %	05 %	06 %	07 %	08 %	09 %	10 %	11 %	12 %	13 %	14 %	15 %	16 %	17 %	18 %	19 %
**Nordic**																															
Finland	52	52	52	53	53	54	55	56	57	58	59	61	62	63	64	65	66	67	68	68	68	69	70	70	71	72	72	73	73	74	74
Denmark	19	19	19	20	20	20	20	19	19	19	19	18	18	17	17	16	16	15	15	15	15	15	14	14	14	14	13	13	13	13	13
Iceland	0	0	0	0	0	0	0	0	0	0	0	0	0	0	0	0	0	0	0	0	0	0	0	0	0	0	1	1	1	1	1
Norway	29	29	28	27	27	26	25	24	24	23	22	21	20	19	19	18	18	17	17	16	16	16	15	15	15	14	14	13	13	13	13
**Other Western**																															
Former Yugoslavia	5	6	6	7	8	11	14	15	16	17	18	19	20	20	21	22	22	23	23	24	24	24	24	25	25	26	26	27	27	28	29
Central & Eastern Europe	53	52	52	51	49	46	44	43	42	41	39	38	37	37	36	35	34	34	33	33	33	33	32	32	33	32	33	33	33	34	34
Western Europe	33	33	34	34	35	34	34	35	35	36	36	37	37	38	38	38	39	39	39	39	40	40	40	39	39	38	38	37	36	35	34
USA, Can, Aus, NZ	9	9	8	8	8	8	7	7	7	7	6	6	6	5	5	5	4	4	4	4	4	4	4	3	3	3	3	3	3	3	3
**Non-Western**																															
Central & Southern America	25	24	24	23	23	22	21	20	20	20	19	19	19	18	18	18	18	18	18	18	18	18	18	18	18	18	18	17	17	17	17
SS Africa	3	3	4	4	4	5	5	5	6	6	6	6	6	6	7	7	7	7	7	8	8	9	9	9	10	10	10	10	10	10	10
Other Middle East	6	6	7	7	7	8	8	8	8	8	8	8	8	9	9	9	9	9	9	9	10	10	10	10	10	10	10	10	10	9	9
Syria	3	3	3	4	4	4	4	5	4	5	5	5	5	5	5	5	5	5	5	5	5	5	4	5	5	6	7	8	9	9	9
Iran & Iraq	14	17	19	20	21	22	23	23	23	24	25	26	27	27	28	27	28	28	29	29	30	30	30	30	29	29	29	29	29	29	30
Turkey	26	25	23	22	21	21	20	19	19	19	19	18	18	17	17	16	16	15	15	14	13	13	12	11	11	10	10	9	9	9	9
Other Asia	12	11	11	10	9	9	9	8	8	8	8	7	7	7	7	7	7	6	6	6	6	6	5	5	5	5	5	4	4	4	4
South East Asia	4	4	4	4	4	4	4	4	4	4	4	4	4	4	4	4	4	4	4	4	4	4	4	4	4	4	4	4	4	5	5
South Asia	6	6	6	6	6	6	6	6	6	6	6	6	6	6	6	7	7	7	7	7	7	7	7	7	7	7	7	7	7	7	7
Rest of the World	1	1	1	1	1	1	1	1	1	1	1	1	1	1	1	1	1	1	1	1	1	1	1	1	1	1	1	1	1	1	1

Notes: percentages are column percentages *within* the three broader parental origin groups.

Source: author's calculations based upon Swedish register collection "Ageing Well"

Table S3. The changing parental age-origin composition of G2 Nordic, Western and non-Western, 1990-2019.

**Second-generation** *(all ages)*						**1990s**										**2000s**										**2010s**					
	89	90	91	92	93	94	95	96	97	98	99	00	01	02	03	04	05	06	07	08	09	10	11	12	13	14	15	16	17	18	19
	%	%	%	%	%	%	%	%	%	%	%	%	%	%	%	%	%	%	%	%	%	%	%	%	%	%	%	%	%	%	%
**Nordic**																															
Finland	66	66	66	66	66	66	67	67	67	67	67	67	66	66	66	66	66	66	66	66	66	65	65	65	65	65	65	65	65	65	65
Denmark	15	15	15	15	15	15	15	15	15	15	15	15	15	15	15	15	15	15	15	15	15	15	15	15	15	16	16	16	16	16	16
Iceland	0	0	1	1	1	1	1	1	1	1	1	1	1	1	1	1	1	1	1	1	1	1	1	1	1	1	1	1	1	1	1
Norway	19	19	18	18	18	18	18	18	18	18	18	18	18	18	18	18	18	18	18	18	18	18	18	18	18	18	19	19	19	19	19
**Other Western**																															
Former Yugoslavia	13	13	14	14	15	16	17	17	18	19	19	20	20	20	21	21	22	22	23	23	24	24	24	25	25	25	25	26	26	26	26
Central & Eastern Europe	29	29	29	29	29	29	28	28	28	28	28	28	28	28	28	28	28	28	28	28	28	28	29	29	29	29	30	30	30	30	30
Western Europe	49	49	49	48	47	47	46	46	45	45	44	44	43	43	43	42	42	41	41	40	40	39	39	39	38	38	37	37	37	36	36
USA, Can, Aus, NZ	9	9	9	9	9	9	9	9	9	9	9	9	9	9	9	9	9	8	8	8	8	8	8	8	8	8	8	8	8	8	8
**Non-Western**																															
Central & Southern America	21	20	20	20	19	19	18	18	17	17	17	17	16	16	16	16	16	16	16	16	15	15	15	15	15	14	14	14	13	13	13
SS Africa	9	10	10	10	11	11	12	12	13	13	14	14	14	14	14	14	13	14	14	14	14	14	15	15	16	16	17	17	17	18	18
Other Middle East	15	16	16	16	16	16	16	16	16	16	16	15	15	15	15	15	15	15	14	14	14	14	14	14	13	13	13	13	13	12	12
Syria	4	4	4	4	5	5	5	5	5	5	5	5	5	5	5	5	5	5	5	5	5	5	5	5	5	5	5	6	7	7	8
Iran & Iraq	9	10	10	11	11	12	13	13	13	14	14	15	15	16	16	16	17	17	18	18	18	19	19	19	19	19	19	19	19	19	19
Turkey	21	20	19	18	17	17	16	16	15	15	15	14	14	14	13	13	13	12	12	12	12	11	11	11	10	10	10	9	9	9	8
Other Asia	5	5	5	5	5	5	4	4	4	4	4	4	4	5	5	5	5	5	5	5	5	5	5	5	5	5	5	4	4	4	4
South East Asia	6	6	6	6	7	7	7	7	7	7	7	7	8	8	8	8	8	8	8	8	8	8	8	8	8	8	8	8	8	8	7
South Asia	9	9	9	8	8	8	8	8	7	7	7	7	7	7	7	7	8	8	8	8	8	8	8	8	8	8	9	9	9	9	9
Rest of the World	1	1	1	1	1	1	1	1	1	1	1	1	1	1	1	1	1	1	1	1	1	1	1	1	1	1	1	1	1	1	1

**Second-generation** *(0-14 years-old)*						**1990s**										**2000s**										**2010s**					
	89	90	91	92	93	94	95	96	97	98	99	00	01	02	03	04	05	06	07	08	09	10	11	12	13	14	15	16	17	18	19
	%	%	%	%	%	%	%	%	%	%	%	%	%	%	%	%	%	%	%	%	%	%	%	%	%	%	%	%	%	%	%
**Nordic**																															
Finland	76	75	74	74	74	73	73	72	72	71	71	70	69	68	67	65	64	62	60	58	57	55	53	52	50	49	47	46	45	44	42
Denmark	12	12	12	12	12	12	12	13	13	13	13	13	14	14	14	15	15	16	17	18	19	20	21	22	23	23	24	24	25	26	26
Iceland	1	1	1	1	1	1	1	1	2	2	2	2	2	2	2	2	2	2	2	3	3	3	3	3	4	4	4	4	4	5	5
Norway	11	12	12	12	13	13	13	14	14	14	14	15	16	16	17	18	19	20	20	21	22	22	23	23	24	24	25	25	26	26	27
**Other Western**																															
Former Yugoslavia	21	21	20	21	23	25	27	28	30	31	32	34	35	36	37	38	39	40	40	40	39	38	37	37	36	36	35	35	34	34	34
Central & Eastern Europe	27	28	28	28	28	28	27	27	27	26	26	26	25	25	25	25	25	25	25	26	27	28	29	30	31	32	33	34	34	35	35
Western Europe	45	44	44	43	41	39	38	36	35	34	33	32	31	30	30	29	28	27	27	27	27	26	26	26	26	26	25	25	25	24	24
USA, Can, Aus, NZ	7	7	8	8	8	8	8	9	9	9	9	9	9	9	9	8	8	8	8	8	8	8	7	7	7	7	7	7	7	7	6
**Non-Western**																															
Central & Southern America	21	21	21	20	19	19	18	17	17	16	16	16	15	15	15	15	14	14	14	14	14	13	13	13	13	13	12	12	11	11	10
SS Africa	9	9	9	10	10	11	12	13	13	14	14	15	15	15	15	15	15	15	15	16	16	16	17	17	18	18	19	20	20	21	21
Other Middle East	15	15	15	16	16	16	16	16	16	16	16	15	15	15	15	15	14	14	14	13	13	13	12	12	12	12	11	11	11	11	11
Syria	4	5	5	5	5	5	5	5	5	6	6	6	6	6	6	6	5	5	5	5	5	5	5	5	5	5	5	6	7	8	9
Iran & Iraq	10	11	11	12	13	14	14	15	16	16	17	17	18	18	19	19	20	20	21	21	22	22	22	23	23	23	22	22	22	21	21
Turkey	22	21	19	18	17	16	15	14	13	13	12	12	11	11	10	10	10	9	9	9	9	8	8	8	7	7	7	6	6	6	5
Other Asia	4	4	4	4	4	4	4	4	4	4	4	4	4	4	4	4	5	5	5	5	5	5	5	5	5	5	5	4	4	4	4
South East Asia	7	7	7	7	8	8	8	8	8	8	8	8	8	8	8	9	9	9	9	9	9	9	9	9	9	9	8	8	8	7	7
South Asia	8	8	8	8	7	7	7	7	7	7	7	7	7	7	7	7	7	7	7	8	8	8	8	9	9	9	9	10	10	10	10
Rest of the World	1	1	1	1	1	1	1	1	1	1	1	1	1	1	1	1	1	1	1	1	1	1	1	1	1	1	1	1	1	1	1

**Second-generation** *(15-39 years-old)*						**1990s**										**2000s**										**2010s**					
	89	90	91	92	93	94	95	96	97	98	99	00	01	02	03	04	05	06	07	08	09	10	11	12	13	14	15	16	17	18	19
	%	%	%	%	%	%	%	%	%	%	%	%	%	%	%	%	%	%	%	%	%	%	%	%	%	%	%	%	%	%	%
**Nordic**																															
Finland	66	67	68	68	69	70	71	72	72	73	74	74	75	75	75	75	76	76	75	75	75	75	74	74	74	73	73	72	72	71	71
Denmark	16	16	15	15	15	15	14	14	14	14	13	13	13	13	12	12	12	12	12	12	12	12	12	13	13	13	13	13	13	13	13
Iceland	0	0	0	0	0	0	0	0	0	0	0	0	0	0	1	1	1	1	1	1	1	1	1	1	1	1	1	1	1	2	2
Norway	18	18	17	16	16	15	14	14	13	13	13	12	12	12	12	12	12	12	12	12	12	12	12	12	13	13	13	13	14	14	14
**Other Western**																															
Former Yugoslavia	10	11	12	13	13	14	15	15	16	17	17	18	19	19	20	20	21	22	22	24	25	26	27	28	29	29	30	31	31	32	33
Central & Eastern Europe	29	28	27	27	27	26	26	26	26	26	26	26	26	26	26	26	26	26	26	26	26	26	26	26	26	26	26	26	26	26	26
Western Europe	55	55	55	55	55	54	54	54	53	53	52	52	51	50	50	49	48	47	46	44	43	41	40	39	38	38	37	36	35	34	34
USA, Can, Aus, NZ	6	6	6	6	5	5	5	5	5	5	5	5	5	5	5	5	5	6	6	6	6	6	7	7	7	7	7	7	8	8	8
**Non-Western**																															
Central & Southern America	18	18	18	18	18	18	19	19	19	19	20	20	20	20	20	20	20	20	20	19	19	18	18	18	17	17	17	17	16	16	16
SS Africa	13	13	12	12	11	11	11	10	10	10	10	10	9	9	9	9	9	9	10	10	10	11	11	12	12	12	12	13	13	13	13
Other Middle East	20	20	20	20	19	19	18	18	17	17	17	16	16	16	16	16	16	16	16	16	16	16	16	16	16	16	16	15	15	15	15
Syria	1	1	1	2	2	2	3	3	3	3	3	3	4	4	4	4	4	5	5	5	5	5	5	5	5	6	6	6	6	6	6
Iran & Iraq	4	4	4	4	4	4	4	4	5	5	5	6	6	7	8	9	10	10	11	12	12	13	13	14	14	15	15	16	16	17	17
Turkey	16	17	19	21	22	23	24	24	24	25	25	24	24	23	23	22	21	20	19	18	17	17	16	16	15	15	14	14	13	13	12
Other Asia	13	12	11	10	9	8	8	7	7	6	6	5	5	5	4	4	4	4	4	4	4	4	4	4	4	4	4	4	4	4	4
South East Asia	1	1	1	1	2	2	2	3	4	4	5	5	5	6	6	6	6	7	7	7	7	8	8	8	8	8	8	8	8	8	8
South Asia	13	12	11	11	11	10	10	10	10	10	10	9	9	9	9	8	8	8	8	7	7	7	7	7	7	7	7	7	7	7	7
Rest of the World	2	2	2	2	2	2	2	2	2	2	2	2	1	1	1	1	1	1	1	1	1	1	1	1	1	1	1	1	1	1	1

Notes: percentages are column percentages *within* the three broader parental origin groups.

Source: author's calculations based upon Swedish register collection "Ageing Well"

Table S3 (cont.) The changing parental age-origin composition of G2 Nordic, Western and non-Western, 1990-2019.

**Second-generation** *(40-64 years-old)*	89	90	91	92	93	94	95	96	97	98	99	00	01	02	03	04	05	06	07	08	09	10	11	12	13	14	15	16	17	18	19
						**1990s**										**2000s**										**2010s**					
	%	%	%	%	%	%	%	%	%	%	%	%	%	%	%	%	%	%	%	%	%	%	%	%	%	%	%	%	%	%	%
**Nordic**																															
Finland	38	39	39	40	41	42	43	44	45	47	48	49	50	52	53	54	56	57	58	59	60	61	63	64	65	66	67	68	69	69	70
Denmark	17	18	18	18	19	19	19	19	19	19	19	19	19	19	19	18	18	18	18	17	17	17	17	16	16	16	16	15	15	15	15
Iceland	0	0	0	0	0	0	0	0	0	0	0	0	0	0	0	0	0	0	0	0	0	0	0	0	0	0	0	0	0	0	0
Norway	44	44	43	42	40	39	38	37	35	34	33	32	31	29	28	27	26	25	24	23	23	22	21	20	19	18	17	17	16	15	15
**Other Western**																															
Former Yugoslavia	0	0	0	0	0	0	0	0	0	0	1	1	1	1	1	2	2	3	4	5	5	6	7	8	10	10	11	12	13	14	14
Central & Eastern Europe	36	37	39	39	40	39	39	38	38	38	37	37	37	37	37	36	36	35	34	34	33	32	32	31	30	29	28	27	27	27	26
Western Europe	34	34	34	34	35	37	38	40	42	43	44	45	46	47	48	49	50	51	51	52	52	53	53	54	54	54	55	55	55	55	54
USA, Can, Aus, NZ	30	28	27	26	25	23	22	21	20	19	18	17	16	15	14	13	12	11	10	10	9	8	8	7	7	6	6	6	6	5	5
**Non-Western**																															
Central & Southern America	27	27	27	26	26	25	25	25	24	24	24	24	24	23	23	22	23	22	22	21	20	20	19	19	18	18	18	18	18	18	19
SS Africa	16	16	16	16	16	16	15	15	15	16	15	15	16	16	15	15	15	15	14	14	14	13	13	13	13	13	13	12	12	11	11
Other Middle East	4	4	4	4	3	4	4	4	5	5	5	6	6	7	7	8	9	11	12	14	15	16	17	18	19	20	20	20	20	19	19
Syria	0	0	0	0	0	0	1	0	0	0	0	0	0	0	0	0	0	0	0	0	0	1	1	1	1	1	1	1	2	2	2
Iran & Iraq	5	4	4	4	4	4	4	4	4	4	4	4	4	4	4	4	5	5	5	4	4	4	4	4	4	4	4	4	4	4	4
Turkey	2	2	3	3	4	4	5	5	6	6	6	5	5	6	6	6	7	7	9	10	11	11	13	14	15	16	17	19	21	22	23
Other Asia	25	25	26	26	27	27	26	26	25	25	25	25	24	24	24	22	21	20	19	18	17	16	15	14	13	13	12	11	10	9	8
South East Asia	2	2	2	1	1	1	1	1	1	1	1	1	1	1	1	1	1	1	1	1	1	1	1	1	1	1	1	1	1	2	2
South Asia	21	20	20	19	19	19	19	19	19	19	20	19	19	19	19	19	19	18	17	17	16	15	15	14	13	13	12	11	11	10	10
Rest of the World	0	0	0	0	0	0	0	0	0	0	0	0	0	1	1	1	1	2	2	2	2	2	2	2	2	2	2	2	2	2	2

**Second-generation** *(65 years-old plus)*	89	90	91	92	93	94	95	96	97	98	99	00	01	02	03	04	05	06	07	08	09	10	11	12	13	14	15	16	17	18	19
						**1990s**										**2000s**										**2010s**					
	%	%	%	%	%	%	%	%	%	%	%	%	%	%	%	%	%	%	%	%	%	%	%	%	%	%	%	%	%	%	%
**Nordic**																															
Finland									30	32	33	35	35	36	37	37	38	39	39	39	39	39	39	39	39	39	39	39	40	41	42
Denmark				*No data*					17	21	21	20	20	20	20	19	19	18	18	18	17	17	16	16	17	17	18	18	18	19	19
Iceland									0	0	0	0	0	0	0	0	0	0	0	0	0	0	0	0	0	0	0	0	0	0	0
Norway									53	47	46	46	45	44	44	44	44	43	43	43	43	44	44	45	44	44	43	42	41	40	39
**Other Western**																															
Former Yugoslavia									0	0	0	0	0	0	0	0	0	0	0	0	0	0	0	0	0	0	0	0	0	0	0
Central & Eastern Europe				*No data*					22	16	16	16	15	15	16	16	16	17	17	18	19	23	26	30	33	36	38	40	40	40	40
Western Europe									36	39	39	39	40	41	41	42	42	42	42	42	41	40	38	37	35	34	33	34	34	35	37
USA, Can, Aus, NZ									42	44	45	45	45	43	43	42	42	41	41	40	39	37	35	33	31	30	28	27	25	24	23
**Non-Western**																															
Central & Southern America									0	0	26	27	32	29	31	32	33	33	30	29	28	28	25	25	26	26	26	26	25	25	24
SS Africa									0	0	0	0	12	13	17	19	17	18	18	18	19	17	17	18	17	17	16	17	16	16	16
Other Middle East									0	0	0	0	0	0	0	0	3	3	4	4	3	4	4	4	5	4	4	4	4	4	4
Syria									0	0	0	0	0	0	0	0	0	0	0	0	0	0	0	0	0	0	0	0	0	0	1
Iran & Iraq				*No data*					0	0	0	0	0	0	0	0	0	0	3	3	3	4	4	5	4	4	4	4	4	3	4
Turkey									0	0	0	0	0	0	0	4	3	3	2	2	2	1	2	2	2	2	3	3	4	4	5
Other Asia									0	0	43	43	32	30	27	23	23	23	21	23	23	23	24	23	24	24	25	25	26	27	27
South East Asia									0	0	0	0	0	0	0	0	0	0	0	0	0	0	0	0	0	1	1	1	1	1	1
South Asia									0	0	30	30	25	29	25	23	20	20	23	22	22	22	23	23	22	22	21	21	20	19	19
Rest of the World									0	0	0	0	0	0	0	0	0	0	0	0	0	0	0	0	0	0	0	0	0	0	0

Notes: percentages are column percentages *within* the three broader parental origin groups.

Source: author's calculations based upon Swedish register collection "Ageing Well"

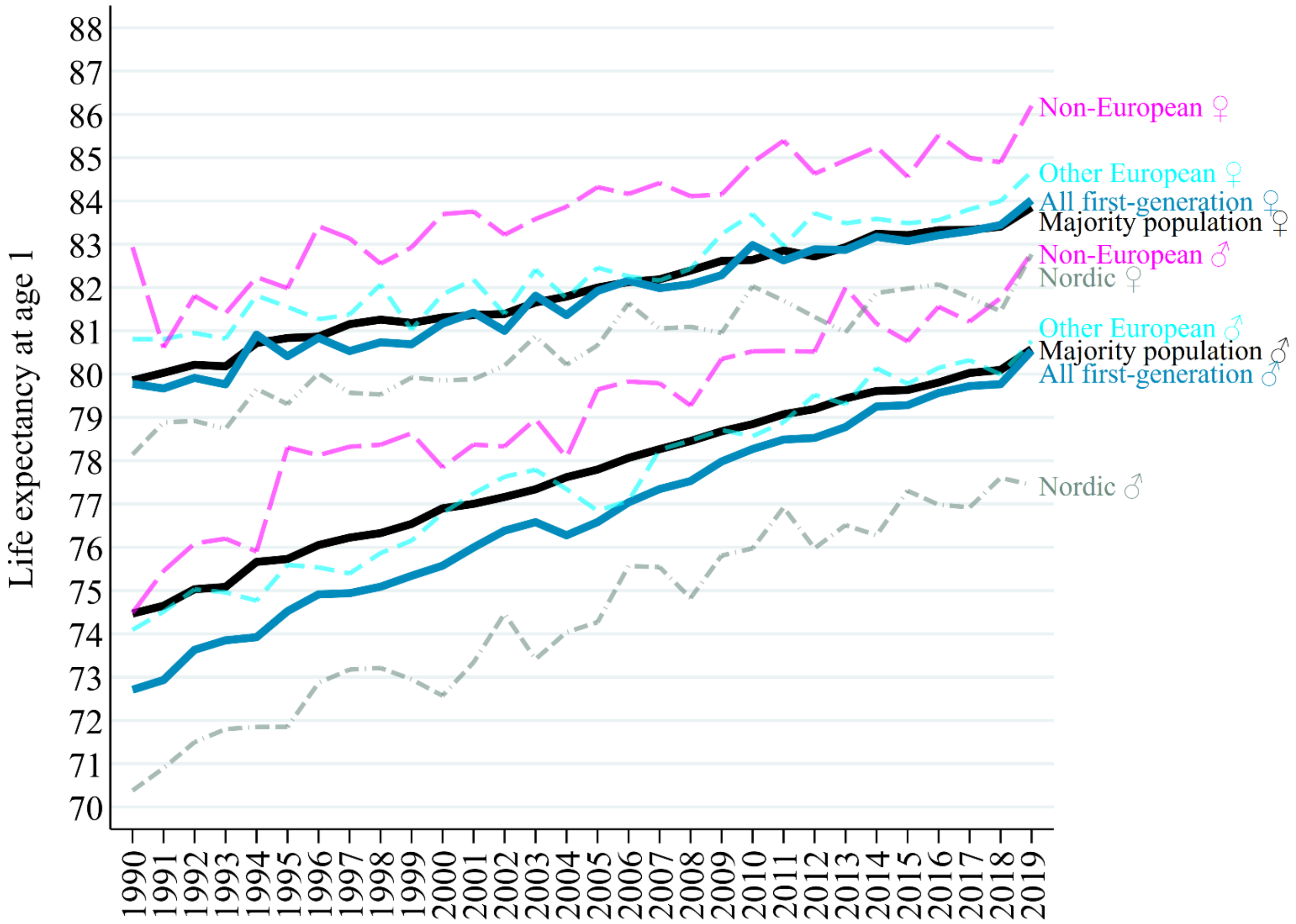

Figure S1. Comparison of life expectancy at birth between first-generation migrants and the majority population between 1990 and 2019.

Source: author's calculations based upon Swedish register collection "Ageing Well"

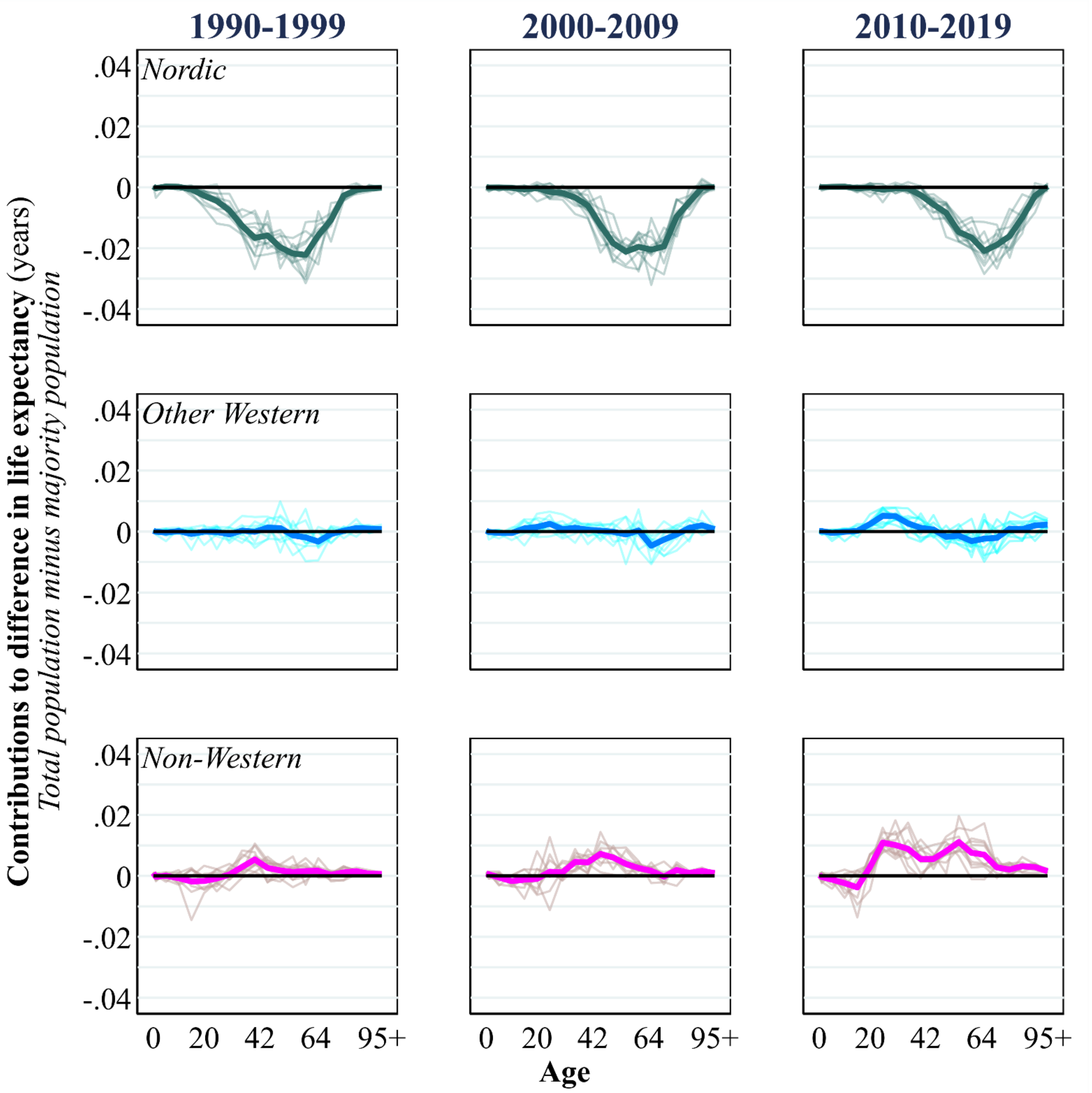

Figure S2. First-generation men, origin by age decompositions of life expectancy differences between total population and total population minus the first and second-generation, 1990-2019.

Source: authors' calculations based upon Swedish register collection "Ageing Well".

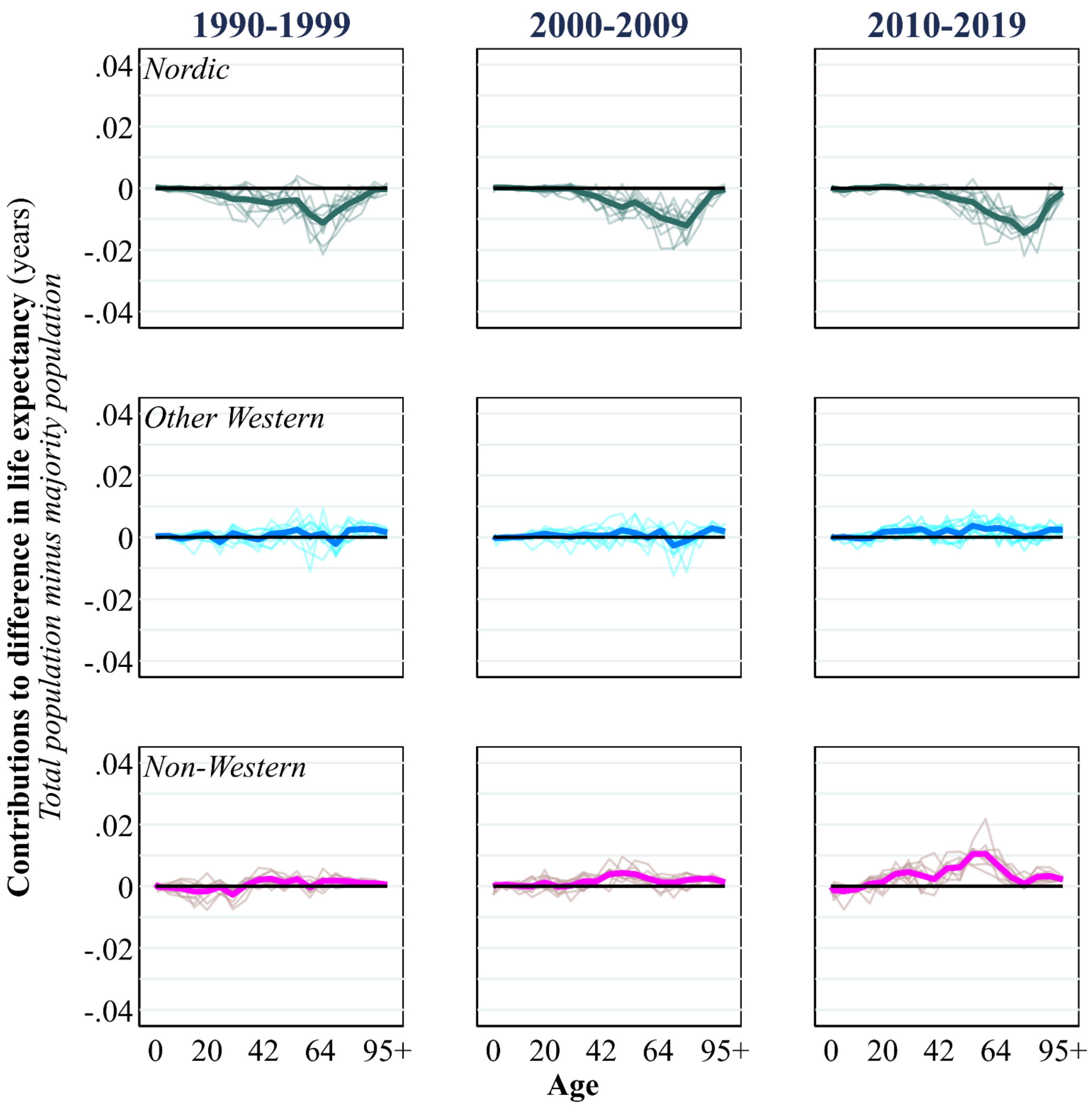

Figure S3. First-generation women, origin by age decompositions of life expectancy differences between total population and total population minus the first and second-generation, 1990-2019.

Source: authors' calculations based upon Swedish register collection "Ageing Well".

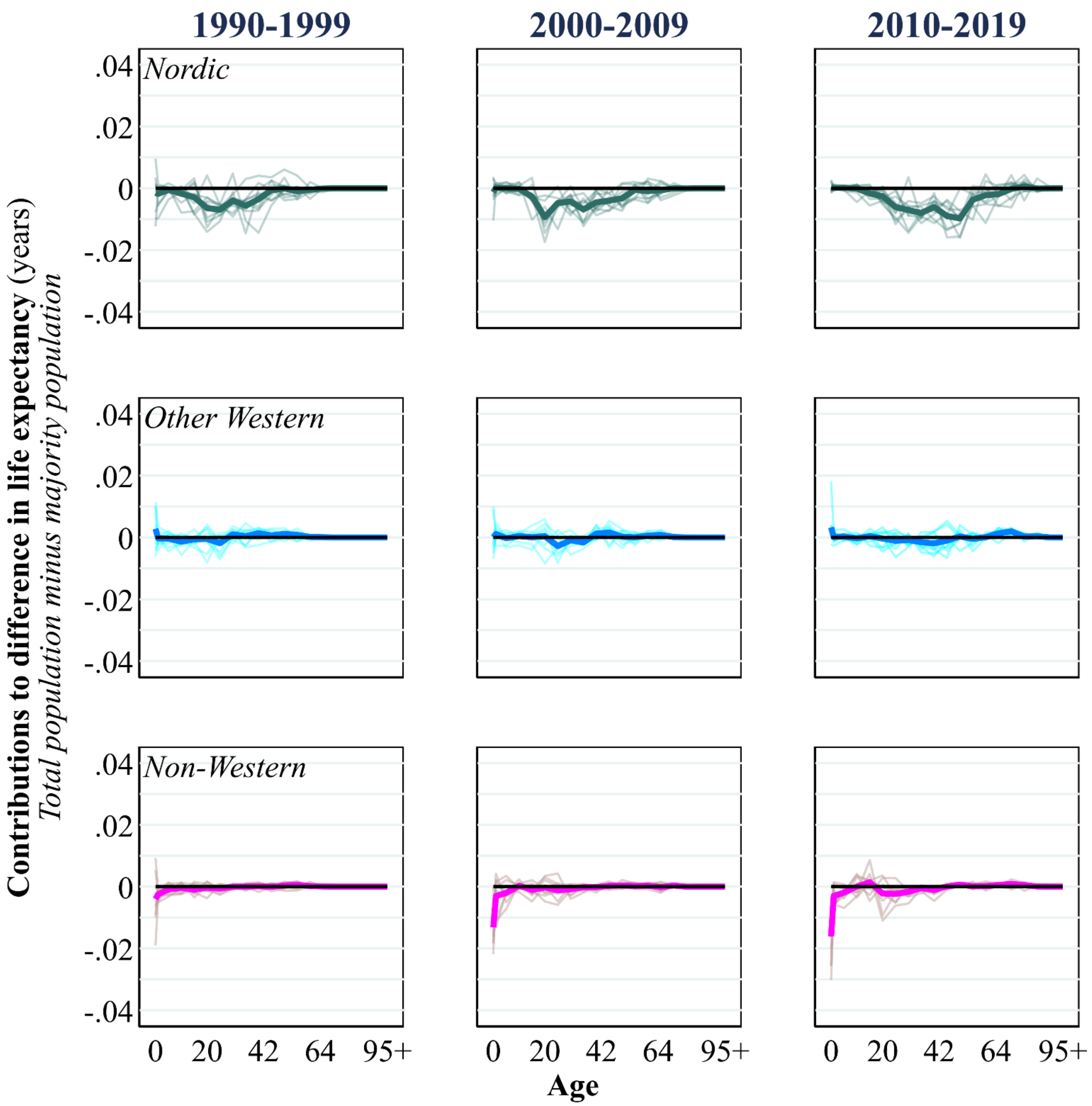

Figure S4. Second-generation men, origin by age decompositions of life expectancy differences between total population and total population minus the first and second-generation, 1990-2019.

Source: authors' calculations based upon Swedish register collection "Ageing Well".

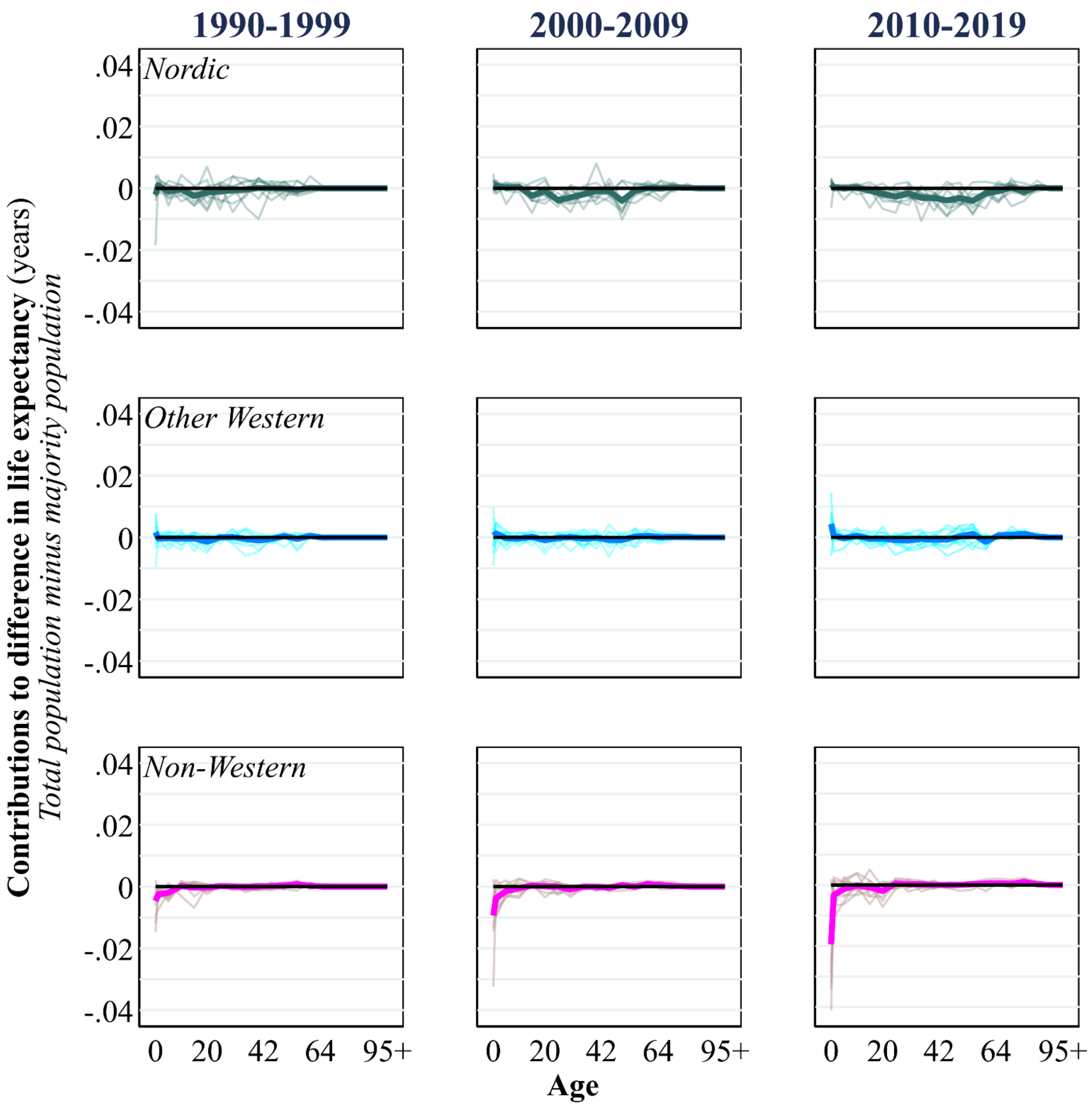

Figure S5. Second-generation women, origin by age decompositions of life expectancy differences between total population and total population minus the first and second-generation, 1990-2019.

Source: authors' calculations based upon Swedish register collection "Ageing Well".

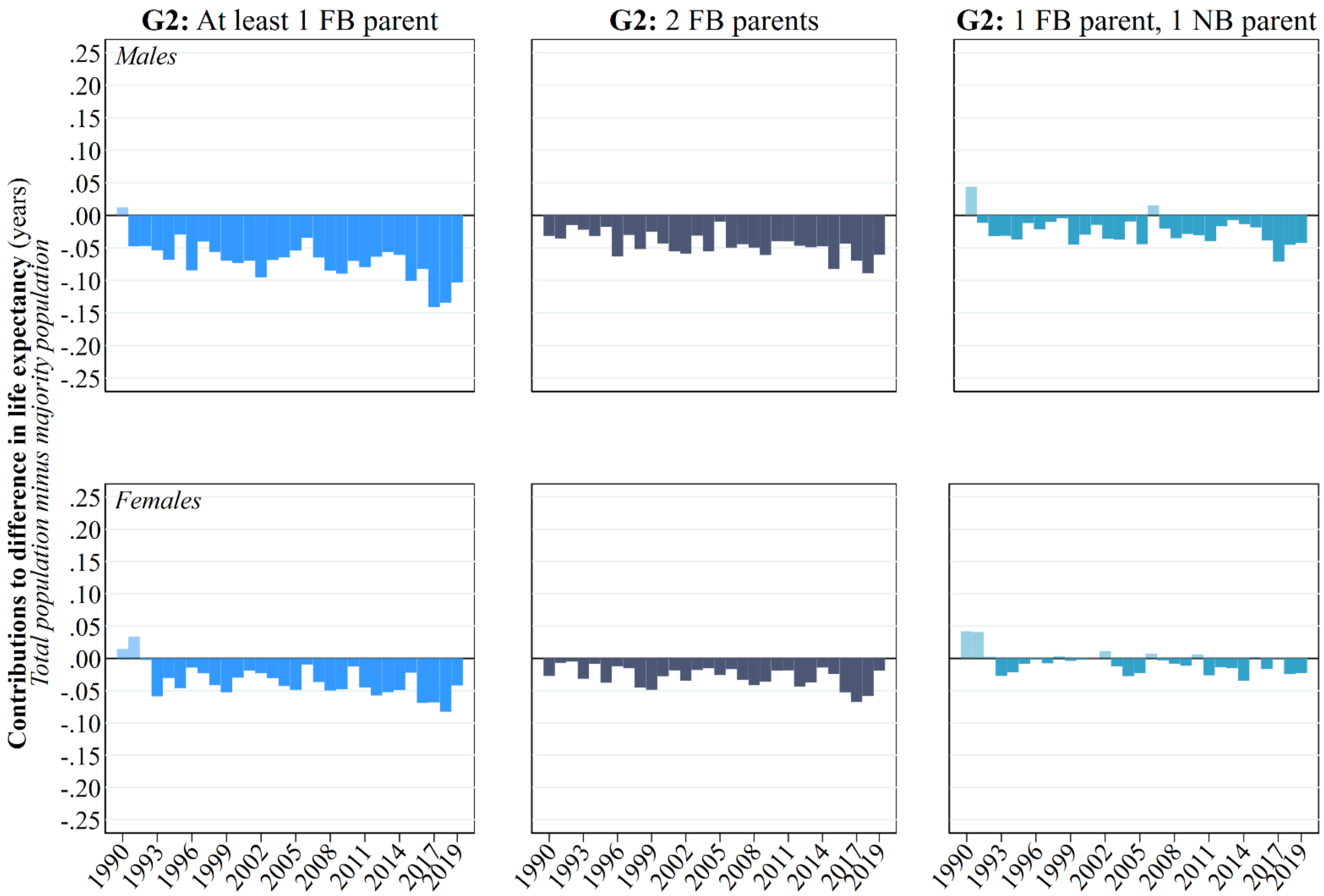

Figure S6. Decomposition of the impact of the second-generation (at least one foreign-born parent) according to whether both parent (G2) or only one parent (G2.5) was foreign-born (FB), 1990-2019.
Source: authors' calculations based upon Swedish register collection "Ageing Well".

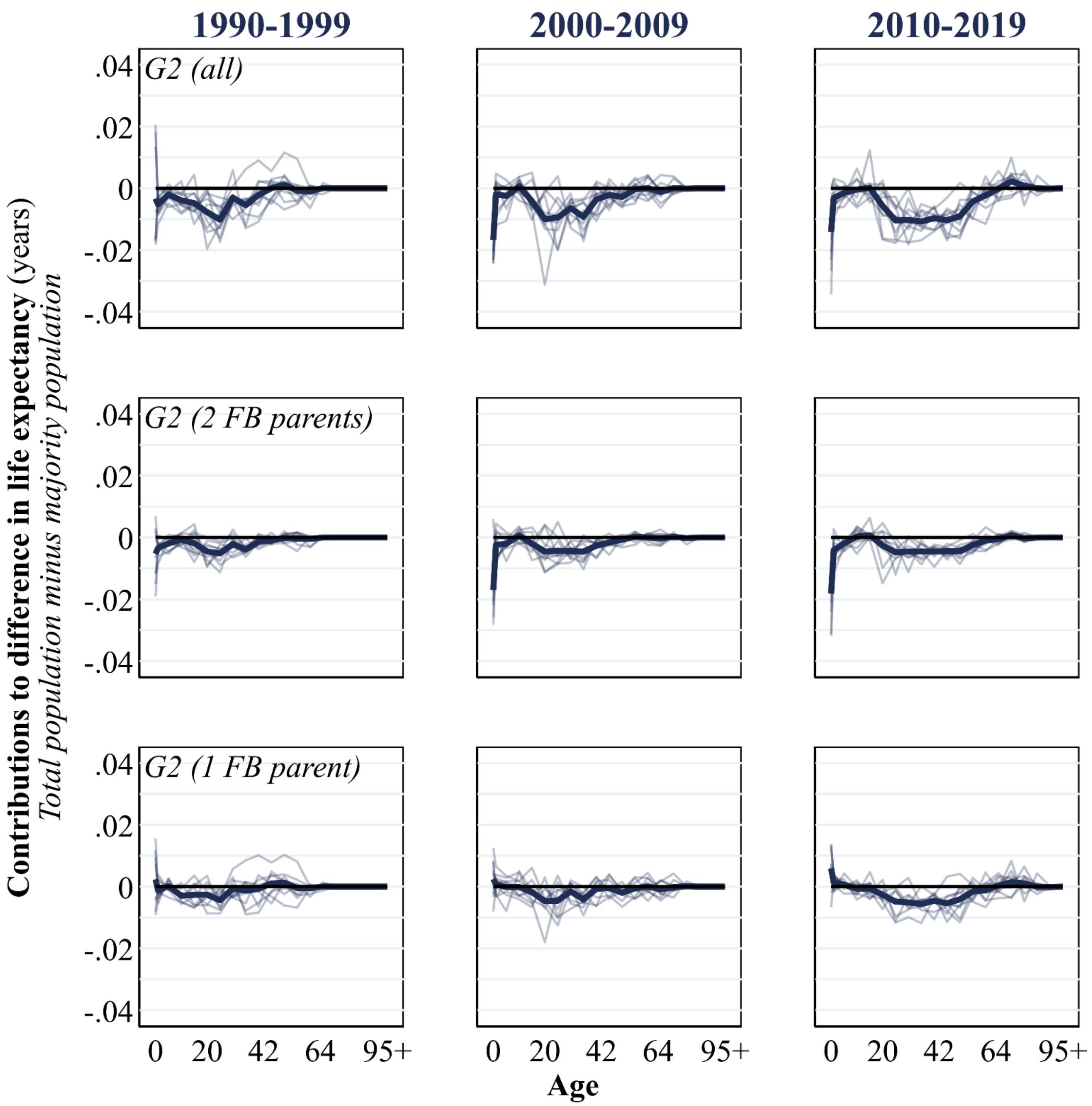

Figure S7. Age decomposition of the impact of second-generation men (at least one foreign-born parent) according to whether both parent (G2) or only one parent (G2.5) was foreign-born (FB), 1990-2019.

Source: authors' calculations based upon Swedish register collection "Ageing Well".

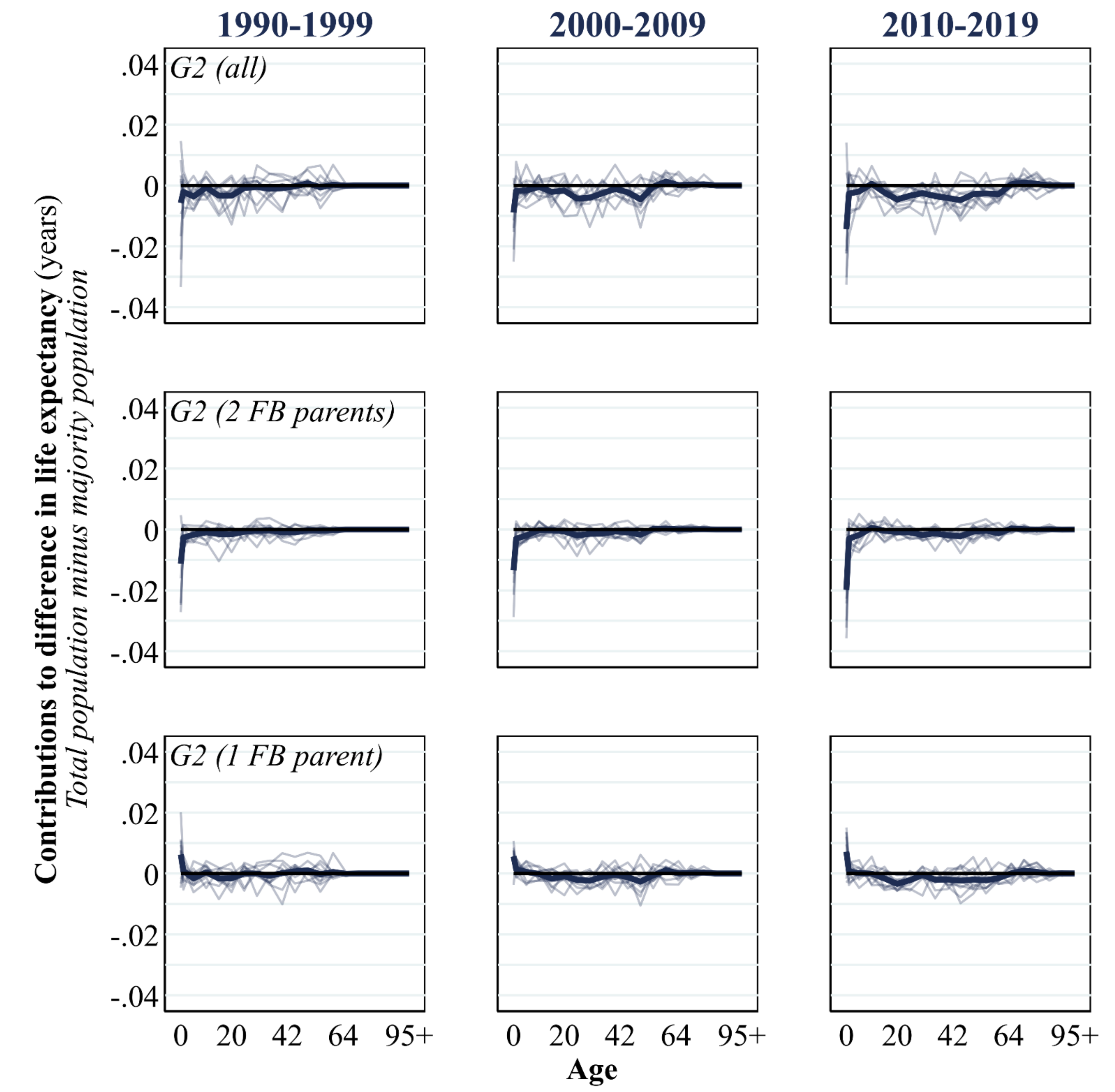

Figure S8. Age decomposition of the impact of second-generation women (at least one foreign-born parent) according to whether both parent (G2) or only one parent (G2.5) was foreign-born (FB), 1990-2019.

Source: authors' calculations based upon Swedish register collection "Ageing Well".
